# Exploring the Impact of Obesity on Progression and Prognosis in Early-Stage Endometrioid Endometrial Carcinoma

**DOI:** 10.1177/26884844251374981

**Published:** 2025-09-05

**Authors:** Jiaxin Wang, Guiping Shen, Huan Jiang, Xinshu Cao, Shenshen Yao, Hua Zheng, Shuying Meng, Zhe Su, Liansheng Tian, Jian Gao, Jun Wang

**Affiliations:** ^1^ Department of Obstetrics and Gynecology, Benxi Central Hospital, Benxi, P.R. China.; ^2^ Department of Clinical Laboratory, Benxi Central Hospital, Benxi, P.R. China.; ^3^ Key Laboratory of Maternal-Fetal Medicine of Liaoning Province, Shenyang, P.R. China.; ^4^ Department of Oncology, Benxi Central Hospital, Benxi, P.R. China.; ^5^ Department of Pathology, Benxi Central Hospital, Benxi, P.R. China.

**Keywords:** endometrioid endometrial carcinoma, BMI, saturated fatty acids, palmitic acid, Mendelian randomization analysis, dietary management

## Abstract

**Background:**

Endometrioid endometrial carcinoma (EEC) is the most prevalent malignancy affecting the female reproductive system, and obesity is a significant risk factor. In this study, we examined the influence of saturated fatty acids (SFAs) on early-stage EEC progression.

**Methods:**

A two-sample Mendelian randomization (MR) analysis using single-nucleotide polymorphisms as instrumental variables was used to assess the potential causal associations among body mass index (BMI), the main components of adipose tissue, and EEC. Clinical data from 231 patients with EEC at Benxi Central Hospital were analyzed according to BMI categories. CCK-8, apoptosis, cell cycle, scratch, and transwell assays were used to examine the biological behavior of Ishikawa cells treated with palmitic acid (PA), the main SFA component. GraphPad Prism v10.2.0 was employed to perform correlation analysis.

**Results:**

MR analysis revealed a statistically causal relationship between BMI and EEC (inverse variance weighted, *p* = 3.573 × 10^−7^). Furthermore, SFAs (inverse variance weighted, *p* = 0.032) and triglycerides (inverse variance weighted, *p* = 0.036) played a notable role in the influence of BMI, and a high BMI was correlated with cervical invasion risk (*p* < 0.001). PA promoted Ishikawa cell proliferation at 24 hours and significantly enhanced migration and invasion at 48 hours.

**Conclusions:**

This research highlights the clinical significance and implications of BMI in the evaluation of poor prognosis in early-stage EEC and the potential role of SFAs in the proliferative, migratory, and invasive abilities of EEC. Our findings emphasize the importance of dietary weight management, particularly for patients with stage I EEC.

## Introduction

Endometrial carcinoma (EC) is the fourth most frequently diagnosed cancer in women worldwide, and its incidence and mortality rates are steadily increasing.^1^ In 2022, 420,242 new cases of EC were reported globally, leading to 97,704 deaths.^2^ EC can be classified into two types according to clinical, endocrine, and epidemiological characteristics: type 1 (endometrioid type), which is hormonally driven, and type 2, which is more aggressive and consists of specialized subtypes such as clear cell and serous carcinomas, which are estrogen-independent.^3^ Although the precise etiology of EC remains unclear, obesity is recognized as one of the most important risk factors for endometrial cancer.^4^ Obesity poses challenges for the diagnosis and treatment of high-risk women, and its contribution to both the incidence and mortality rates of EC is remarkably significant.^5^ Observational studies indicate that obesity, defined as a body mass index (BMI) between 30.0 and 35.0 kg/m^2^, is linked to a 2.6-fold higher risk of developing EC, whereas severe obesity (BMI ≥35.0 kg/m^2^) corresponds to a 4.7-fold higher risk.^6^ Studies utilizing BMI as a measure of obesity have demonstrated a direct correlation between higher BMI and EC incidence.^7^ Endometrioid endometrial carcinoma (EEC), a malignant epithelial tumor with endometrioid differentiation, accounts for more than 80% of ECs and takes on the glandular columnar morphology of normal endometrium.^8^ However, the specific component of adipose tissue that plays a key role in promoting EEC progression is currently unclear.

To further explore the impact of obesity on patients with EEC, we used single-nucleotide polymorphisms (SNPs) from genome-wide association studies (GWAS) as instrumental variables (IVs) in a two-sample Mendelian randomization (MR) analysis to evaluate the effect of obesity on EEC. Our results reveal an obvious causal relationship between BMI and EEC. Based on the clinical data, we found that patients with Fédération Internationale de Gynécologie etd’Obstétrique (FIGO) stage II EEC had a significantly higher BMI than those with stage I EEC. Furthermore, the main component of adipose tissue was used as the exposure variable, with BMI as the outcome variable in a detailed MR analysis. The results revealed a significant correlation between saturated fatty acids (SFAs) (the main components of triglycerides) and BMI. This study was designed to investigate whether an imbalance of SFA increases the malignant potential of early-stage ECC. Specifically, the objective was to assess changes in different biological behaviors of Ishikawa cells following exposure to palmitic acid (PA), the primary SFA found in high-fat diets (HFD),^9^ and to provide a theoretical foundation for research into the poor prognosis observed in patients with early-stage EEC consuming an HFD.

## Materials and Methods

### MR analysis

All MR analyses were conducted with the two-sample MR package in R, in which the causal effects of an exposure on an outcome were assessed according to summary statistics from the GWAS database (https://gwas.mrcieu.ac.uk/). SNPs were used as IVs. To evaluate the significance of the results, we applied multiple methods, including inverse-variance-weighted MR (IVW-MR, the key analytical method), MR-Egger regression, simple mode, weighted median, and weighted mode. The sensitivity and robustness of the findings were confirmed using heterogeneity, pleiotropy, and leave-one-out sensitivity tests. A *p*-value threshold of *p* < 5 × 10^−8^ was applied for the GWAS analysis.

### Clinical data collection

We collected data from 231 patients with EEC admitted to the Central Hospital of Benxi between January 2019 and December 2024. We analyzed the differences in BMI among the patients on the basis of general and clinicopathological characteristics, with a focus on those with FIGO stage I–II EEC. All patients included in the study had no history of other cancers, had not received neoadjuvant treatments, such as chemotherapy or radiotherapy, before surgery, and provided signed informed consent.

### Immunohistochemistry

EEC tissues were fixed in paraformaldehyde (4%), dehydrated, cleared, embedded in paraffin, sectioned into 3-μm slices, and prepared for immunohistochemical staining with Ki-67 primary antibody (ZM-166, ZSGB-BIO, China) following standard protocols. For immunohistochemistry, sections were dewaxed and subjected to antigen retrieval, followed by exposure to 3% hydrogen peroxide and 5% serum for 30 minutes. The sections were then incubated with primary antibodies overnight at 4°C. A negative control without the primary antibody was included, and nonspecific background staining was not detected. The sections were subsequently exposed to a biotin-conjugated secondary antibody and stained with DAB. High-magnification images (200×) were captured using the MShot Image Analysis System software.

### Cell culture

Ishikawa cells (Biospecies, China) were cultured in MEM containing 15% FBS and 1% NEAA, and maintained in a sterile environment at 37°C with 5% CO_2_ and high humidity. To examine the potential impact of SFAs on the biological behavior of EECs, Ishikawa cells were treated with PA (APEN2456, APExBIO, USA), whereas control cells were treated with an equivalent volume of BSA solution.

### Cell proliferation assay

Ishikawa cells were cultivated in 96-well plates (8 × 10³ cells/well), with six replicates per group. After 24 hours, the cells were treated according to the experimental conditions. Two hours before measurement, 10 μL of Cell Counting Kit-8 (CK10001, Glpbio, China) reagent was added, and the optical density (OD) was read at 450 nm with a Bio-Rad microplate reader.

Colony formation assays were conducted to evaluate the ability of individual cells to form clones, which were identified as distinct colonies. Ishikawa cells were grown in 6-well plates (5 × 10^2^ cells/well) with three replicates per group. After 24 hours, the cells were treated with either PA or BSA solution according to their respective group assignments and incubated in a sterile environment. Cell status was checked every two days, and colony-forming cells were harvested from 7 to 10 days based on the cell growth rate. The number of colonies and cells within each colony was observed and recorded under a microscope (MI52-N, Mshot, China).

### Cell cycle and apoptosis analyses

Ishikawa cells were grown in 6-well plates, with three replicates per group. After 24 h, the cells were exposed to PA or control solutions. Cell migration was observed and photographed using an inverted microscope at 0, 24, and 48 hours post-treatment, after which an initial scratch was made.

For apoptosis detection, Ishikawa cells treated with PA or the control were resuspended in binding buffer. Next, annexin V-FITC (5 μL) and propidium iodide (PI: 5 μL) were added. The cells were incubated in the dark for 15 minutes, and flow cytometry was used to determine the number of apoptotic cells.

### Migration and invasion

Ishikawa cells were grown in 6-well plates, with three replicates per group. After 24 hours, the cells were exposed to PA or control solutions. Cell migration was observed and photographed using an inverted microscope at 0, 24, and 48-hours post-treatment, after which an initial scratch was made.

Matrigel- or uncoated Transwell chambers were placed in 24-well plates. The lower chambers were filled with 500 μL of culture medium containing 15% FBS, and 200 μL of single-cell suspension (5 × 10^4^ cells/well) was added to the upper chambers. The plates were then incubated in a sterile incubator at 37°C and 5% CO_2_ for 48 hours. After incubation, the samples were collected and rinsed thrice with PBS. The cells were fixed with 4% formaldehyde for 20 minutes and stained with 0.1% crystal violet for 10 minutes. Nonmigrating or noninvading cells were gently removed. All fields of view were photographed at 200× magnification and counted for statistical analysis.

### Statistical analysis

In this study, data analysis was performed using R Studio v1.1.46 with R software v4.1.1 and GraphPad Prism v10.2.0. The main method of MR analysis is IVW analysis, and the odds ratio (OR) estimate is either greater than 1 or less than 1. The data obtained from the two-group comparisons are presented as the mean ± SEM. Normality testing was conducted using the Shapiro–Wilk test, whereas the homogeneity of variance was examined using Levene’s test. Two-tailed unpaired Student’s *t*-tests were used to analyze group comparisons, and Pearson’s chi-square tests were used to examine the associations between age, lesion size, and BMI. Jordon’s index was used to calculate the cutoff value for age. Statistical significance was set at *p* < 0.05.

## Results

### MR analysis

**FIG. 1.**
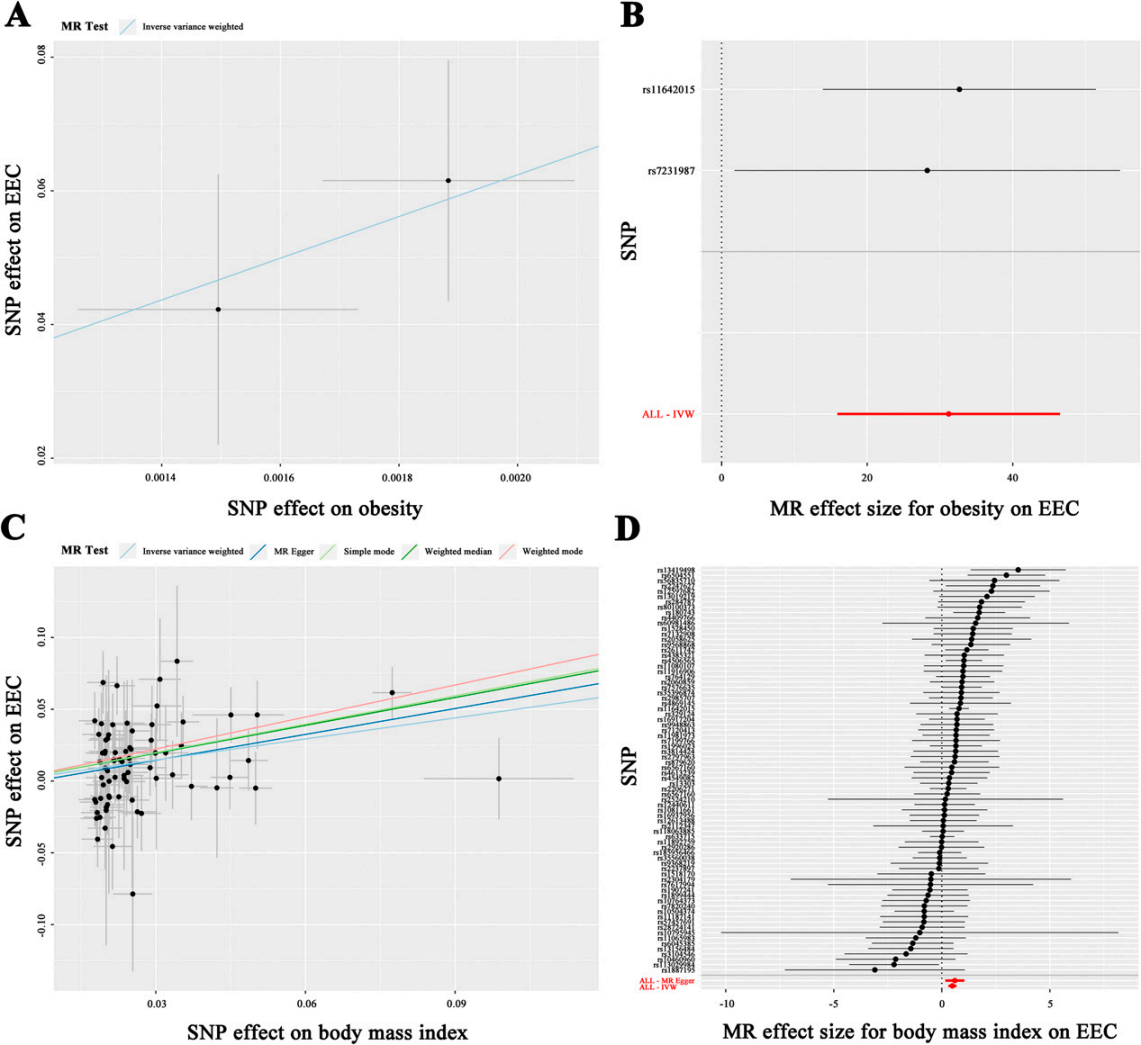
MR analyses of the causal effect of obesity and BMI on EEC. **(A)** Scatter plot illustrating the role of obesity in EEC. Each black dot denotes an SNP, with the *x*-axis indicating the SNP’s estimate on obesity and the y-axis indicating its estimate on EEC risk, including standard error bars. The line slopes represent causal estimates derived from the inverse variance weighted method. **(B)** Forest plot displaying the estimated risk of obesity on EEC. **(C)** Scatter plot showing the impact of BMI on EEC, with line slopes indicating causal estimates from five different methods. **(D)** Forest plot presenting the estimated risk of BMI on EEC. BMI, body mass index; EEC, endometrioid endometrial carcinoma; MR, Mendelian randomization; SNP, single-nucleotide polymorphism.

To investigate the impact of obesity and the main components of adipose tissue on EEC, we performed MR analysis to identify causal variables closely associated with EEC. We performed a summary statistical analysis using data from the GWAS dataset (ID: ukb-b-15541), which included 463,010 obese individuals, and a BMI-related dataset (ID: ebi-a-GCST90018727) comprising 163,835 cases as exposure factors. For the outcome variables, we used EEC-related data from another dataset (ID: ebi-a-GCST006465), comprising 54,884 cases. Two SNPs associated with obesity were extracted for IVW-MR analysis, yielding an OR of 3.540 × 10^13^ (95% confidence interval [CI]: 7.990 × 10^6^ to 1.568 × 10^20^, *p* = 6.455 × 10^−5^), indicating a significant correlation between obesity and increased EEC risk (Fig. 1A–B, Supplementary Table S1). Additionally, 76 SNPs related to BMI were identified, and univariate MR analysis was conducted using five different methods to predict genetic associations. All five methods demonstrated an obvious association between BMI and high EEC risk (IVW: OR: 1.630, 95% CI: 1.350–1.967, *p* = 3.573 × 10^−7^; MR-Egger: OR: 1.824, 95% CI: 1.173–2.838, *p* = 0.009; weighted median: OR: 1.344, 95% CI: 0.921–1.960, *p* = 2.672 × 10^−6^; simple mode: OR: 1.934, 95% CI: 1.093–3.421, *p* = 0.026; weighted mode: OR: 2.102, 95% CI: 1.465–3.016, *p* = 1.300 × 10^−4^) (Fig. 1C, D, Table 1). Our findings support a potential link between obesity and the risk of EEC. Moreover, we discovered that beyond serving as a key measure of obesity, BMI may also be a crucial factor in assessing EEC risk.

**Table 1.** Mendelian Randomization Analysis of EEC and BMI

Method	No. of SNP	OR	95% CI	*p*-Value
Inverse variance weighted	76	1.630	1.350–1.967	**3.573 × 10^−7^**
MR-Egger	76	1.824	1.173–2.838	**0.009**
Weighted median	76	1.344	0.921–1.960	**2.672 × 10^−6^**
Simple mode	76	1.934	1.093–3.421	**0.026**
Weighted mode	76	2.102	1.465–3.016	**1.300 × 10^−4^**

Boldface type indicates a statistically significant difference (*p* < 0.05).

BMI, body mass index; CI, confidence interval; EEC, endometrioid endometrial carcinoma; OR, odds ratio; SNP, single-nucleotide polymorphism.

**FIG. 2.**
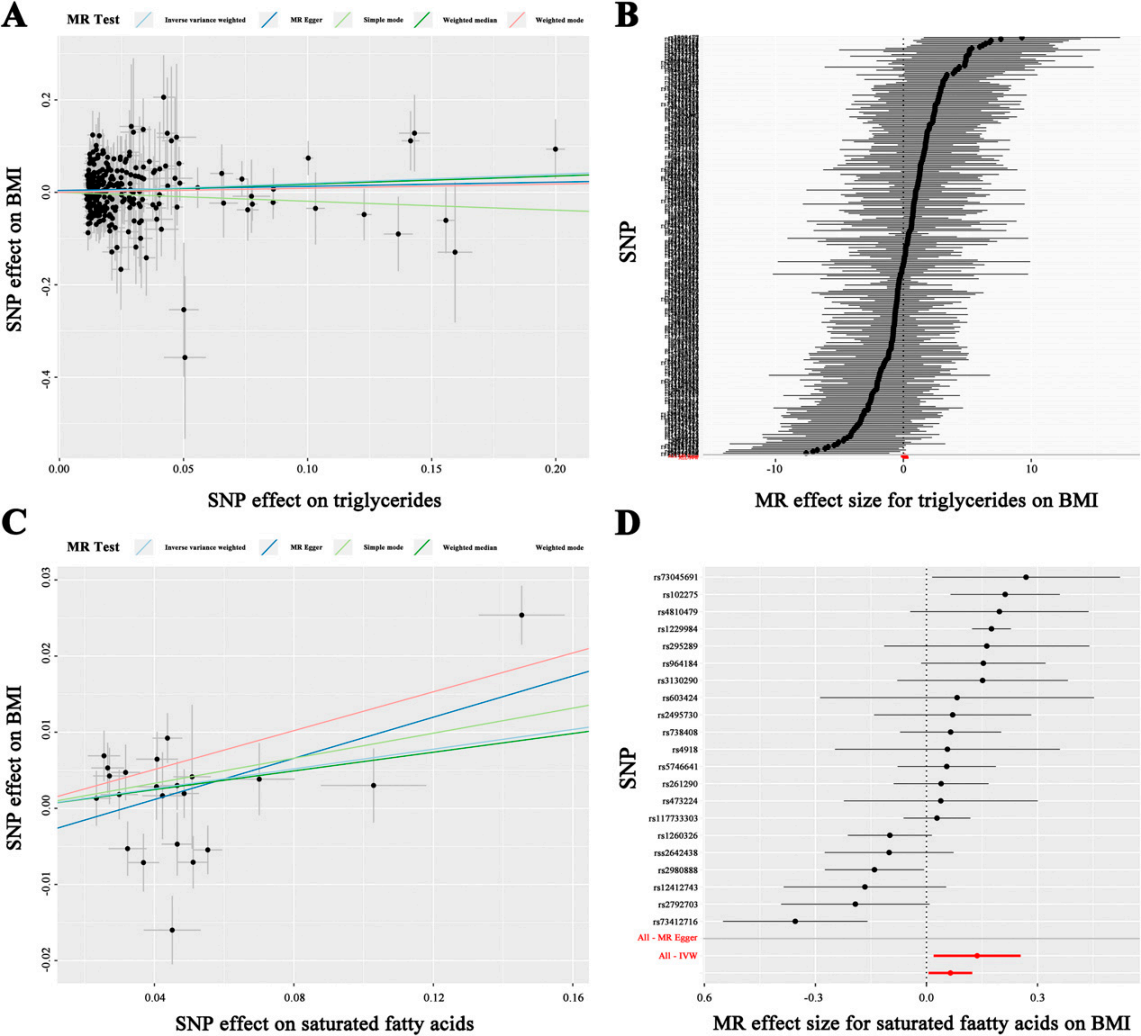
MR analyses of the causal effect of triglycerides and SFAs on BMI. **(A)** Scatter plot illustrating the role of triglycerides in BMI. **(B)** Forest plot displaying the estimated risk of triglycerides on BMI. **(C)** Scatter plot showing the impact of SFAs on BMI. **(D)** Forest plot presenting the estimated risk of SFAs on BMI. SFAs, saturated fatty acids.

To further explore the potential causal associations between the major components (triglycerides, GWAS ID: ieu-b-111,441,016 cases; SFAs, GWAS ID: ebi-a-GCST90092981, 115,006 cases; monounsaturated fatty acids, GWAS ID: met-d-MUFAs, 114,999 cases; polyunsaturated fatty acids, GWAS ID: ebi-a-GCST90092940, 115,006 cases; total cholesterol, GWAS ID: ebi-a-GCST90018974, 344,278 cases; high-density lipoprotein, GWAS ID: ebi-a-GCST90018956, 315,133 cases; low-density lipoprotein, GWAS ID: ebi-a-GCST90018961, 343,621 cases; total phospholipids, GWAS ID: ebi-a-GCST90092991, 115,082 cases) of adipose tissue and EEC, we conducted an MR analysis with the major components of adipose tissue as the exposure and BMI as the outcome variable. The results revealed that triglycerides (IVW: OR: 1.216, 95% CI: 1.013–1.460, *p* = 0.036) and SFAs (IVW: OR: 1.067, 95% CI: 1.006–1.132, *p* = 0.032) were strongly associated with increased BMI (Fig. 2, Tables 2, 3). However, monounsaturated fatty acids (IVW: OR: 1.110, 95% CI: 0.905–1.361, *p* = 0.316), polyunsaturated fatty acids (IVW: OR: 0.892, 95% CI: 0.709–1.124, *p* = 0.333), total phospholipids (IVW: OR: 1.030, 95% CI: 0.838–1.266, *p* = 0.777), total cholesterol (IVW: OR: 1.157, 95% CI: 0.960–1.393, *p* = 0.126), low-density lipoprotein (IVW: OR: 1.102, 95% CI: 0.930–1.305, *p* = 0.262), and high-density lipoprotein (IVW: OR: 0.979, 95% CI: 0.842–1.138, *p* = 0.781) were not significantly correlated with BMI increase (Supplementary Figs. S1–Supplementary Figure S2, Supplementary Table S2).

**Table 2.** Mendelian Randomization Analysis of BMI and Triglycerides

Method	No. of SNP	OR	95% CI	*p*-Value
Inverse variance weighted	268	1.216	1.013–1.460	**0.036**
MR-Egger	268	1.094	0.824–1.453	0.533
Weighted median	268	1.192	0.887–1.603	0.244
Simple mode	268	0.825	0.419–1.621	0.576
Weighted mode	268	1.093	0.824–1.451	0.536

Boldface type indicates a statistically significant difference (*p* < 0.05).

BMI, body mass index; SNP, single-nucleotide polymorphism; OR, odds ratio; CI, confidence interval.

**Table 3.** Mendelian Randomization Analysis of BMI and Saturated Fatty Acids

Method	No. of SNP	OR	95% CI	*p*-Value
Inverse variance weighted	21	1.067	1.006–1.132	**0.032**
MR-Egger	21	1.147	1.020–1.290	**0.034**
Weighted median	21	1.061	0.998–1.128	0.060
Simple mode	21	1.096	0.967–1.243	0.168
Weighted mode	21	1.134	1.058–1.215	**0.002**

Boldface type indicates a statistically significant difference (*p* < 0.05).

BMI, body mass index; SNP, single-nucleotide polymorphism; OR, odds ratio; CI, confidence interval.

### Clinical data analysis

**FIG. 3.**
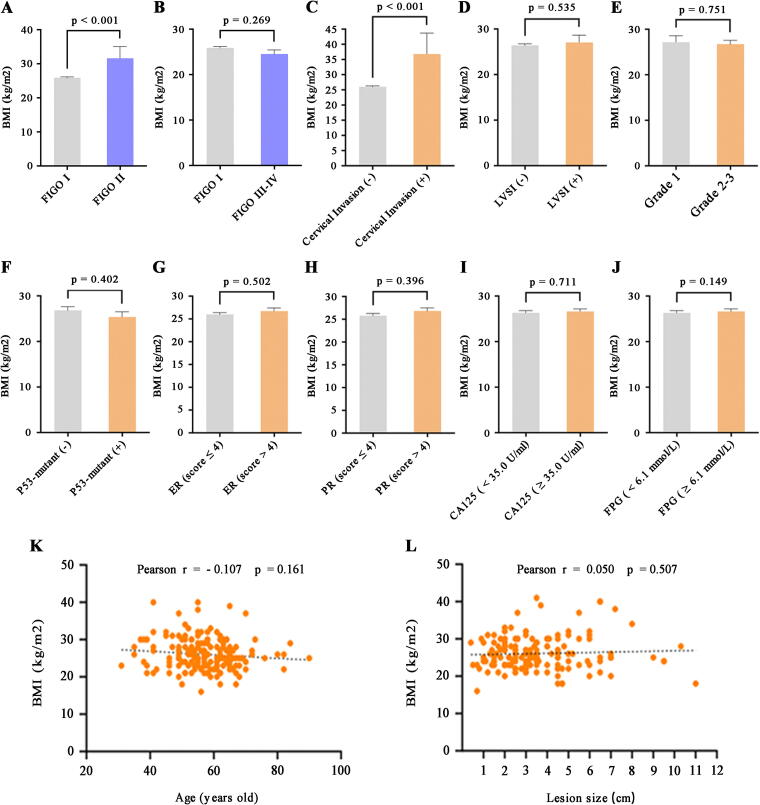
Clinical data analyses of patients with EEC. **(A)** Comparison of clinical parameters between patients with EEC in FIGO stage II and stage I groups revealed a significant difference in BMI (31.647 ± 13.547 vs. 25.874 ± 4.195, *p* < 0.001). **(B)** The BMI of patients with EEC in the FIGO stage III–IV group (24.500 ± 3.026) was slightly lower compared with the FIGO stage I group (25.874 ± 4.195, *p* = 0.269). **(C–J)** Significant differences in BMI were observed between patients with cervical invasion and those without (*p* < 0.001). However, no obvious differences were noted for LVSI (*p* = 0.535), grade (*p* = 0.751), P53 mutation status (*p* = 0.402), ER (*p* = 0.502), PR (*p* = 0.396), CA125 (*p* = 0.711), and FPG (*p* = 0.149) among clinical subgroups of patients with EEC at FIGO stage I–II. *p* < 0.05 indicates statistical significance between the corresponding subgroups, as evidenced by the unpaired Student’s t-test. **(K–L)** The correlation between BMI and age (Pearson *r* = −0.107; *p* = 0.161) and lesion size (Pearson r = 0.050; *p* = 0.507) in patients with EEC at FIGO stage I-II showed no significant associations. CA125, cancer antigen 125; ER, estrogen receptor; FIGO, Fédération Internationale de Gynécologie etd’Obstétrique; FPG, fasting plasma glucose; LVSI, lymphovascular space invasion; PR, progesterone receptor.

In our analysis of BMI and clinical data among patients with EEC, we identified significant differences in BMI among patients at various disease stages. Notably, patients with stage II disease had significantly higher BMIs than those with stage I disease (31.647 ± 13.547 vs. 25.874 ± 4.195, *p* < 0.001) (Fig. 3A, Supplementary Table S3). However, no obvious difference in BMI was noted between patients with stage III/IV and stage I disease (24.500 ± 3.149 vs. 25.874 ± 4.195, *p* = 0.269) (Fig. 3B). Further analysis revealed that among patients with stage I/II disease, those with cervical invasion had significantly higher BMIs than those without cervical invasion (36.875 ± 18.065 vs. 25.982 ± 4.213, *p* < 0.001) (Fig. 3C). Although lymphovascular space invasion (LVSI)-positive patients had slightly higher BMIs than LVSI-negative patients, the difference was not significant (27.077 ± 9.746 vs. 26.391 ± 4.479, *p* = 0.535) (Fig. 3D). Our findings demonstrate that elevated BMI may be a key indicator of the progression of endometrioid carcinoma to stage II (especially stage IIA). While there were no significant differences in the other clinical characteristics in relation to BMI in patients with early-stage EEC, certain trends emerged. Patients with high-grade early-stage endometrioid carcinoma and P53 mutations tended to have lower BMIs (Fig. 3E, F). In contrast, higher BMIs were associated with elevated levels of progesterone receptor (PR), estrogen receptor (ER), cancer antigen 125 (CA125), and fasting plasma glucose (FPG) (Fig. 3G–J). Additionally, a negative correlation was observed between BMI and age, whereas a positive correlation emerged between BMI and larger lesion size in patients with early stage EEC (Fig. 3K, L). These findings suggest a complex interplay between obesity and cancer progression and highlight the significance of BMI as an indicator of early stage EEC.

### PA-induced changes in the biological behavior of Ishikawa cells

**FIG. 4.**
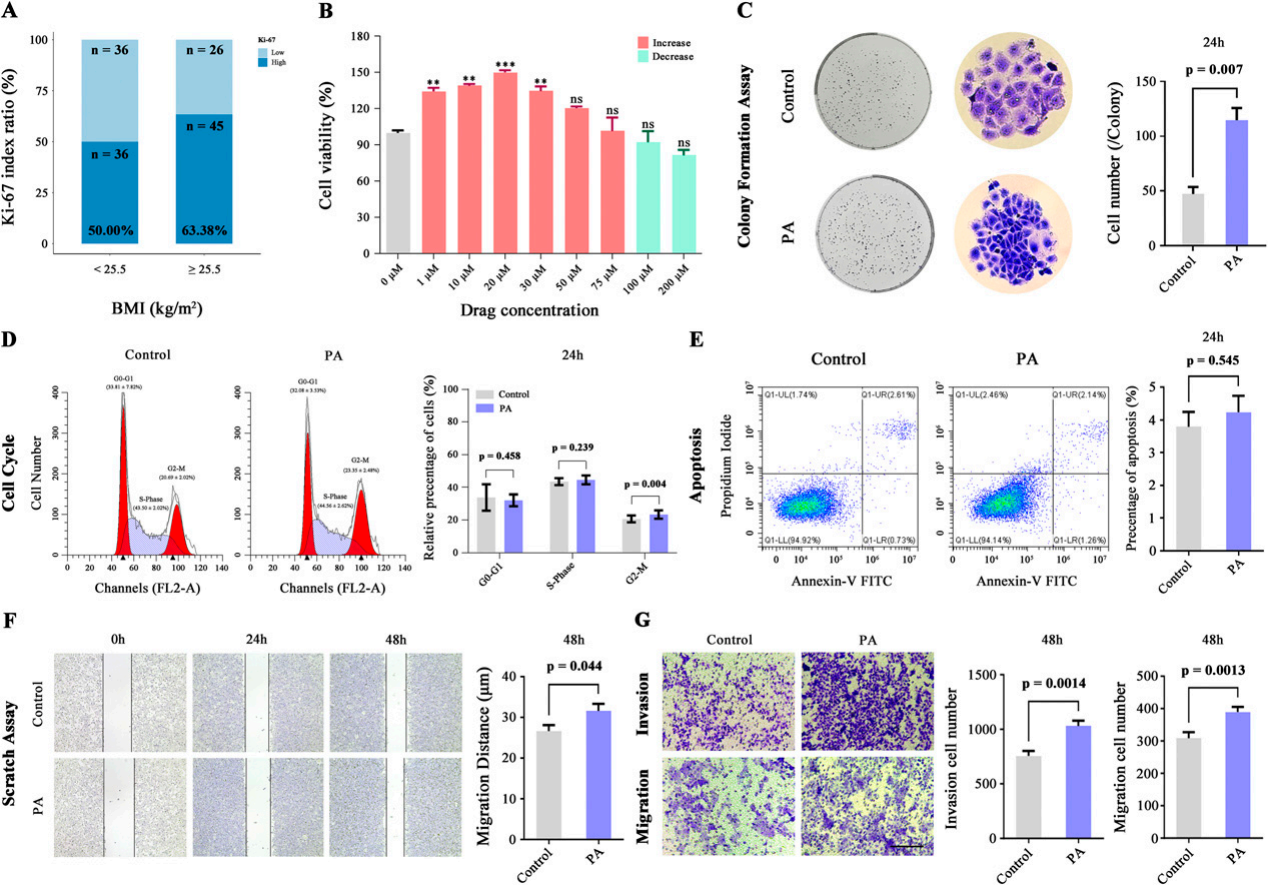
PA-induced changes in the biological behavior of Ishikawa cells. **(A)** The Ki-67 immunohistochemical assay demonstrates the proportional difference in the percentage of patients with Ki-67 expression equal to or exceeding the mean value (40%) between the low-risk group (BMI <25.5 kg/m²) and the high-risk group (BMI ≥25.5 kg/m²), with proportions of 50.00% and 63.38%, respectively. **(B)** Cell proliferation in Ishikawa cells exposed to various concentrations of PA for 24 hours. Data are presented as means ± SEM. A two-tailed Student’s *t-test* was employed to evaluate statistical significance, with **p < 0.01 and ***p < 0.001 comparing each concentration to 0 μM controls. **(C)** Colony formation assay of Ishikawa cells exposed to 20 μM PA for 48 hours showed an increased number of cells per colony. **(D)** Analysis of cell cycle distribution in Ishikawa cells exposed to 20 μM PA for 24 hours revealed average percentages of 32.08 ± 3.53% in G0-G1 phase, 44.56 ± 2.62% in S phase, and 23.35 ± 2.48% in G2-M phase. In contrast, cells exposed to 0 μM PA had distributions of 33.81 ± 7.82% in G0-G1 phase, 43.50 ± 2.02% in S phase, and 20.69 ± 2.02% in G2-M phase. **(E)** Quantitative analysis of Ishikawa cell apoptosis *via* Annexin V/PI staining, comparing control and cells exposed to 20 μM PA for 24 hours. **(F)** Scratch assay of Ishikawa cells exposed to 20 μM PA, compared to controls (0 μM PA). **(G)** Transwell migration and invasion assays of Ishikawa cells exposed to 20 μM PA for 48 hours, with or without Matrigel. Images represent four fields of view, with a scale bar of 50 μm. The relative levels of invasive and migratory cells in the culture supernatant were quantified with Image J software. All data are presented as means ± SD of independent biological triplicates. Ki-67, Kiel-67; PA, palmitic acid; PI, propidium iodide.

**FIG. 5.**
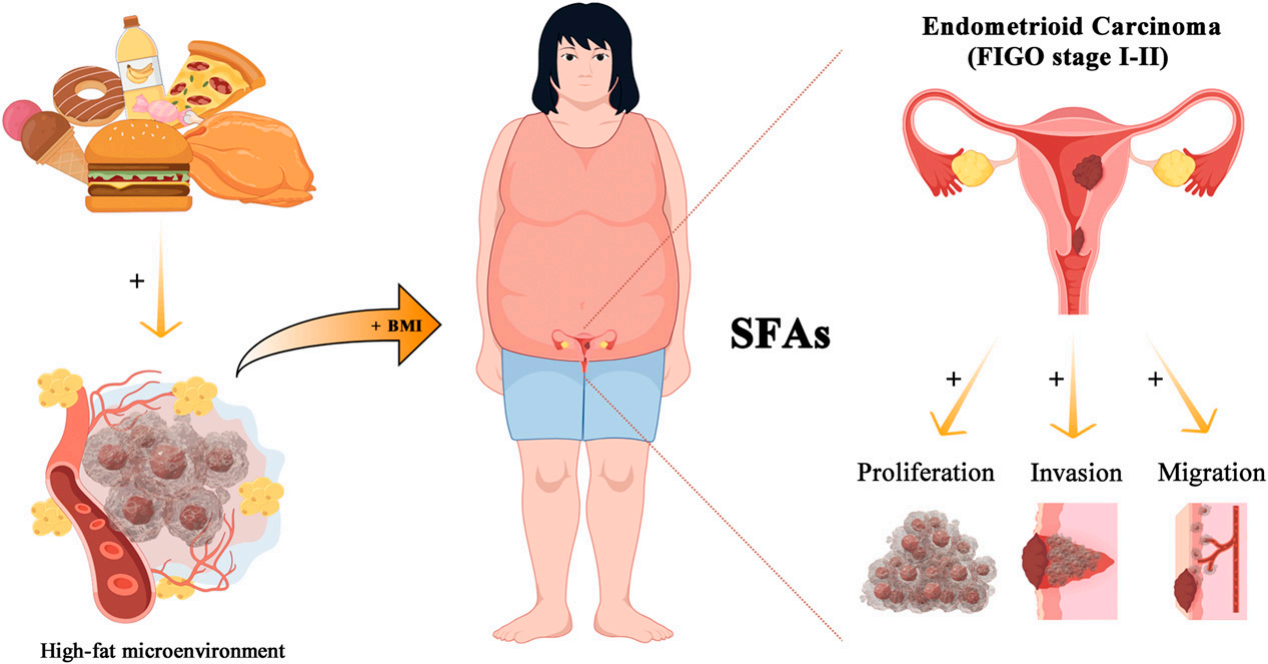
Mechanism diagram of this study. Mechanistic pathways linking obesity-induced high-fat microenvironment, mediated through saturated fatty acids, to enhanced proliferation, migration, and invasion in endometrioid endometrial carcinoma cells.

Among the 231 patients with stage I/II EEC, 143 displayed positive Ki-67 staining *via* immunohistochemistry (with no missing values). Using ROC curve analysis and Youden’s index, the best cutoff point for predicting the risk of occurrence of FIGO stage II was a BMI value of 25.5 kg/m^2^ (80.00% sensitivity, 53.00% specificity) (Supplementary Fig. S3A). Among these patients, the median Ki-67 index (40%) aligns with the findings of Kosmas et al.^10^ Notably, in patients with BMI ≥25.5 kg/m^2^, 63.38% had Ki-67 expression levels exceeding 40% compared to 50.00% in patients with BMI <25.5 kg/m^2^ (Fig. 4A, Supplementary Fig. S3B, Supplementary Table S3), and patients with FIGO stage II EEC showed a similar proliferative trend compared to stage I patients (Supplementary Fig. S3C), suggesting that obesity may enhance the proliferative capacity of early stage EEC. Based on the abovementioned correlation of BMI with the proliferative capacity of early stage EEC and to explore whether SFAs contribute to the malignant biological behavior of EEC, we exposed Ishikawa cells to different concentrations of PA. The results indicated that 20 μM PA had the strongest ability to induce the proliferation of Ishikawa cells (Fig. 4B, Supplementary Fig. S3D). The results of the cell colony formation assay demonstrated that compared with untreated cells, cells treated with 20 μM PA for 24 hours formed significantly larger colonies with a higher cell count than that of the untreated group (114.667 ± 15.752 vs. 47.267 ± 9.301, *p* = 0.007) (Fig. 4C). Moreover, 24 hours post-treatment, the proportion of cells in the G2-M phase was notably higher (23.354 ± 2.478 vs. 20.687 ± 2.016, *p* = 0.004), whereas those in the G0–G1 (32.085 ± 3.530 vs. 33.811 ± 7.822, *p* = 0.458) and S phases (44.562 ± 2.622 vs. 43.498 ± 2.017, *p* = 0.239) remained unchanged (Fig. 4D). Additionally, no obvious difference in apoptosis rate was found between PA-treated and untreated cells (4.237 ± 0.707 vs. 3.790 ± 0.643, *p* = 0.545) (Fig. 4E). Interestingly, after 48 hours of treatment with PA, a significant increase in the cell migration distance was observed in the scratch assays (31.587 ± 6.161 vs. 26.609 ± 5.275, *p* = 0.044) (Fig. 4F), along with an increased number of cells migrating through the Transwell chambers, both with the Matrigel coating (1029.250 ± 221.242 vs. 755.200 ± 145.196, *p* = 0.0014) and without the coating (389.742 ± 86.680 vs. 308.233 ± 101.944, *p* = 0.0013) (Fig. 4G). During this time, a notable increase in the proportion of cells in the G2-M phase was observed (20.797 ± 0.922 vs. 19.151 ± 2.190, *p* = 0.015), along with a decrease in the percentage of cells in the G0–G1 phase (38.011 ± 1.345 vs. 39.527 ± 1.727, *p* = 0.015). However, no obvious difference was found in the proportion of cells in the S phase (41.207 ± 1.778 vs. 41.320 ± 0.985, *p* = 0.837) (Supplementary Fig. S4). These findings indicate that BMI is a valid indicator of poor EEC prognosis in patients with early-stage disease and that SFAs may play an adverse role in the development of early-stage EEC, which may be closely related to the high-fat microenvironment caused by obesity (Fig. 5).

## Discussion

EC is a prevalent gynecological malignancy with a global incidence rate of approximately 5.9%, as reported by the Global Cancer Observatory, and this rate continues to increase worldwide.^11^ Although molecular classification of EC using the TCGA database provides valuable insights for risk stratification and has been integrated into the latest National Comprehensive Cancer Network guidelines,^12–13^ the 1983 histological classification criteria remain widely used in clinical practice because of their long-standing acceptance and familiarity among practitioners.^14^ According to data from the International Agency for Research on Cancer, obesity is strongly related to an increased incidence of EEC and is associated with an increased risk of cancer-related mortality.^15–16^ In a retrospective study of women with early stage EEC, those with morbid obesity were observed to have a higher mortality rate than those with normal BMI.^17^ These findings indicate that obesity is not only a high-risk factor for the onset of EEC but also significantly influences its development and progression.

Obesity typically occurs when energy intake exceeds energy expenditure, leading to excess fat being stored in the fat cells.^18^ Adipocytes are specialized cells that store energy in the form of lipids and play critical roles in metabolic regulation and energy homeostasis.^19^ Triglycerides, the dominant form of fat stored in adipose tissue, function as primary energy reservoirs within the body and are capable of undergoing biochemical transformations to meet energy demands in various physiological states.^20^ Growing evidence suggests that elevated triglyceride levels may contribute to the risk and progression of EC by promoting carcinogenesis through altered lipid metabolic pathways.^21–22^ In the present study, we found that an elevated BMI may play a prominent role in EEC progression from stage I to II, and that there is a significant positive correlation between triglycerides, particularly their key components, SFAs, and the risk of developing an elevated BMI on the basis of MR and clinical data analysis. Furthermore, using PA, the main component of SFAs, we confirmed that SFAs may exacerbate the proliferative, migratory, and invasive abilities of EEC. These findings reveal a potential pathway by which obesity leads to disease progression in patients with EEC.

The overconsumption of energy-dense foods (such as unprocessed and processed meats, salty snacks, or pastries) can increase the levels of SFAs in the body, and elevated levels of SFAs are associated with an increased risk of cancer.^23–24^ During the progression of malignant tumors, cell proliferation, migration, and invasion occur at distinct and unsynchronized stages. Typically, proliferation precedes other processes, allowing the tumor to establish and expand. As cancer undergoes further genetic and molecular alterations, cells may subsequently gain the ability to migrate and invade the adjacent tissues.^25^ PA is the predominant SFA, accounting for 20%–30% of total fatty acids in the human body, and serves as one of the major energy sources for the body.^26–27^ D. Yam et al.^28^ reported significantly elevated plasma PA levels in patients with EC compared with healthy controls. Moreover, serum PA levels have been consistently shown to correlate positively with patients’ BMI.^29^ A key aspect of PA’s physiological role is the maintenance of its concentration and distribution across different lipid classes, which requires precise metabolic regulation. However, this homeostatic control can be persistently disrupted under physiopathological conditions such as obesity and cancer.^30^ Studies have shown that malignant tumor cells take up serum PA to meet proliferation energy demands and potentially promote metastasis.^31^ Paradoxically, excessively high concentrations of PA may exert inhibitory effects on cell proliferation, including in endometrioid carcinoma cells.^32^ In this *in vitro* study, 20 μM PA effectively stimulated the proliferation of Ishikawa endometrioid carcinoma cells. This concentration aligns with the PA range (3–50 μM) used by Kwan et al. to promote malignant melanoma cell proliferation.^33^ These findings suggest that PA may more effectively support malignant cell growth and energy supply at relatively low concentrations. Whether HFD-induced obesity promotes tumor progression by disrupting the dynamic balance of PA in the endometrioid carcinoma microenvironment remains uncertain and is a key focus of our ongoing research. As an estrogen-dependent subtype, changes in estrogen levels contribute significantly to the progression of EEC.^34^ According to a recent report, PA can elevate aromatase and estrogen expression levels in ovarian granulosa cell-extracellular matrix.^35^ Additionally, PA has been shown to promote testosterone-to-estrogen conversion *via* aromatase in astrocytes.^36^ This mechanism may also occur in non-ovarian tissues of patients with early-stage EC affected by obesity. Estrogen binds to estrogen receptor-alpha (ERα), which activates the classical E2/ERα signaling pathway, resulting in crosstalk with the insulin/insulin receptor signaling pathway, leading to the activation of the downstream PI3K/AKT/mTOR and MAPK signaling pathways.^37–38^ These pathways are critical for the initiation and progression of EEC, as they promote cell proliferation and survival at multiple levels.^39–40^ Furthermore, SFAs have been implicated in the activation of specific inflammatory signaling cascades, such as the nuclear factor kappa-light-chain-enhancer of activated B cells pathway, which can increase the expression of genes involved in cell survival, proliferation, and angiogenesis, thereby supporting cancer growth and resistance to apoptosis.^41–42^ In addition, SFAs can modulate immune responses, potentially affecting the ability of the immune system to detect and eliminate cancer cells. The overconsumption of dietary SFAs is considered a contributing factor to obesity and can reduce the number and antitumor activity of CD8+ T cells within tumors, compete for lipid molecules, and accelerate tumor growth.^43^ These pathways may also play functional roles in the process of progression. Nonetheless, further high-quality studies are required to confirm these associations. The findings of the present study could aid the development of dietary recommendations for EEC prevention and clinical strategies. These strategies could incorporate diet-related interventions based on BMI to modulate the levels of SFAs in patients, potentially improving the outcomes of EEC when combined with first-line treatment.

However, this study has several limitations: (1) the sample size of patients with a BMI ≥ of 35 kg/m^2^ and advanced EEC (FIGO stage III–IV) was relatively small; (2) data on blood lipid levels were not included; and (3) the molecular and cellular mechanisms underlying the influence of SFAs on early stage EEC progression were not fully explored, as relevant data are still being collected for further investigation.

## Conclusion

The results of our study indicated that SFA-induced alterations in the body microenvironment may influence the progression and prognosis of early-stage endometrioid carcinoma. These findings provide a theoretical basis for recommending that patients with EEC, particularly those with stage I disease, adopt a balanced diet and manage their weight as a part of clinical care.

## Data Availability

All data are available in the article or supplementary materials.

## Abbreviations Used

BMI: Body mass index
BSA: Bovine serum albumin
CA125: Cancer antigen 125
EEC: Endometrioid endometrial carcinoma
ER: Estrogen receptor
GWAS: Genome-wide association studies
HFD: High-fat diets
IVs: Instrumental variables
IVW: Inverse variance weighted
LVSI: Lymphovascular space invasion
MR: Mendelian randomization
OD: Optical density
OR: Odds ratio
PA: Palmitic acid
PBS: Phosphate buffered saline
PR: Progesterone receptor
ROC: Receiver operating characteristic
SFA: Saturated fatty acid
SNP: Single-nucleotide polymorphisms
