# Supplementary figures and images for: Exploring the Impact of Obesity on Progression and Prognosis in Early-Stage Endometrioid Endometrial Carcinoma
Source: Womens Health Rep (New Rochelle). 2025 Sep 5;6(1):803–15. doi: 10.1177/26884844251374981 (PMC12528850; doi:10.1177/26884844251374981)

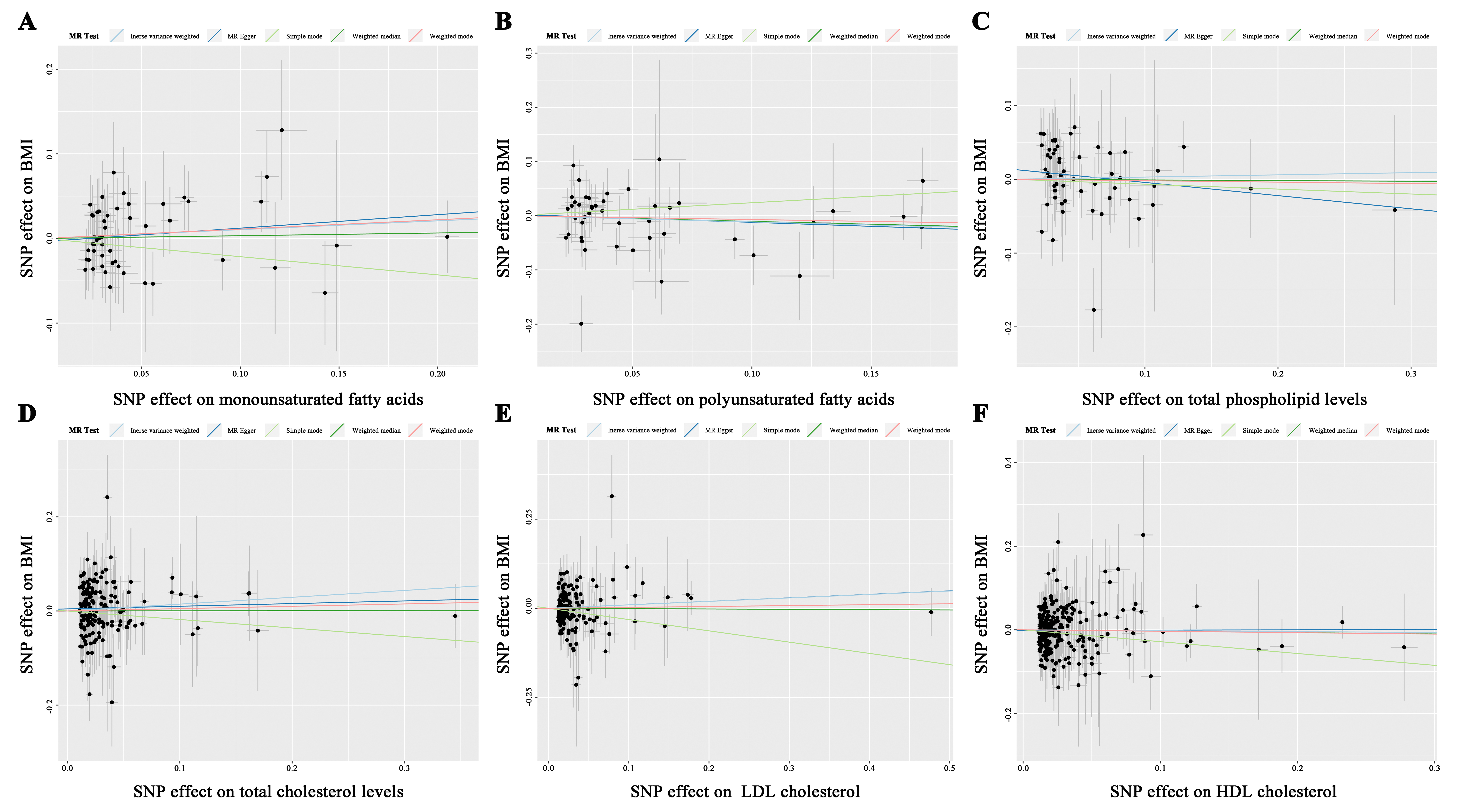

Supplement: Supplementary Figure S1 [file 26884844251374981_supplementary_figure_s1.tif]

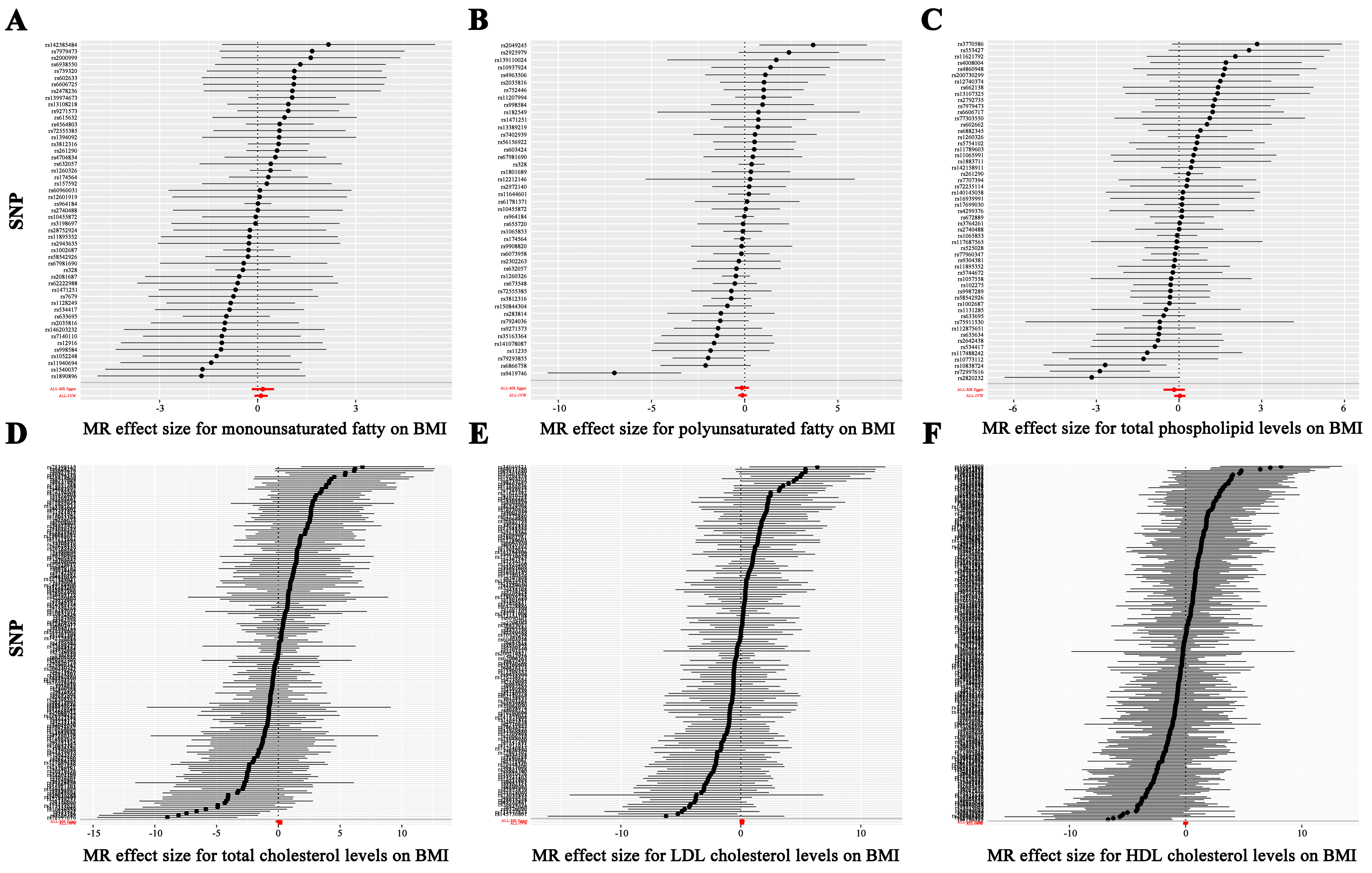

Supplement: Supplementary Figure S2 [file 26884844251374981_supplementary_figure_s2.tif]

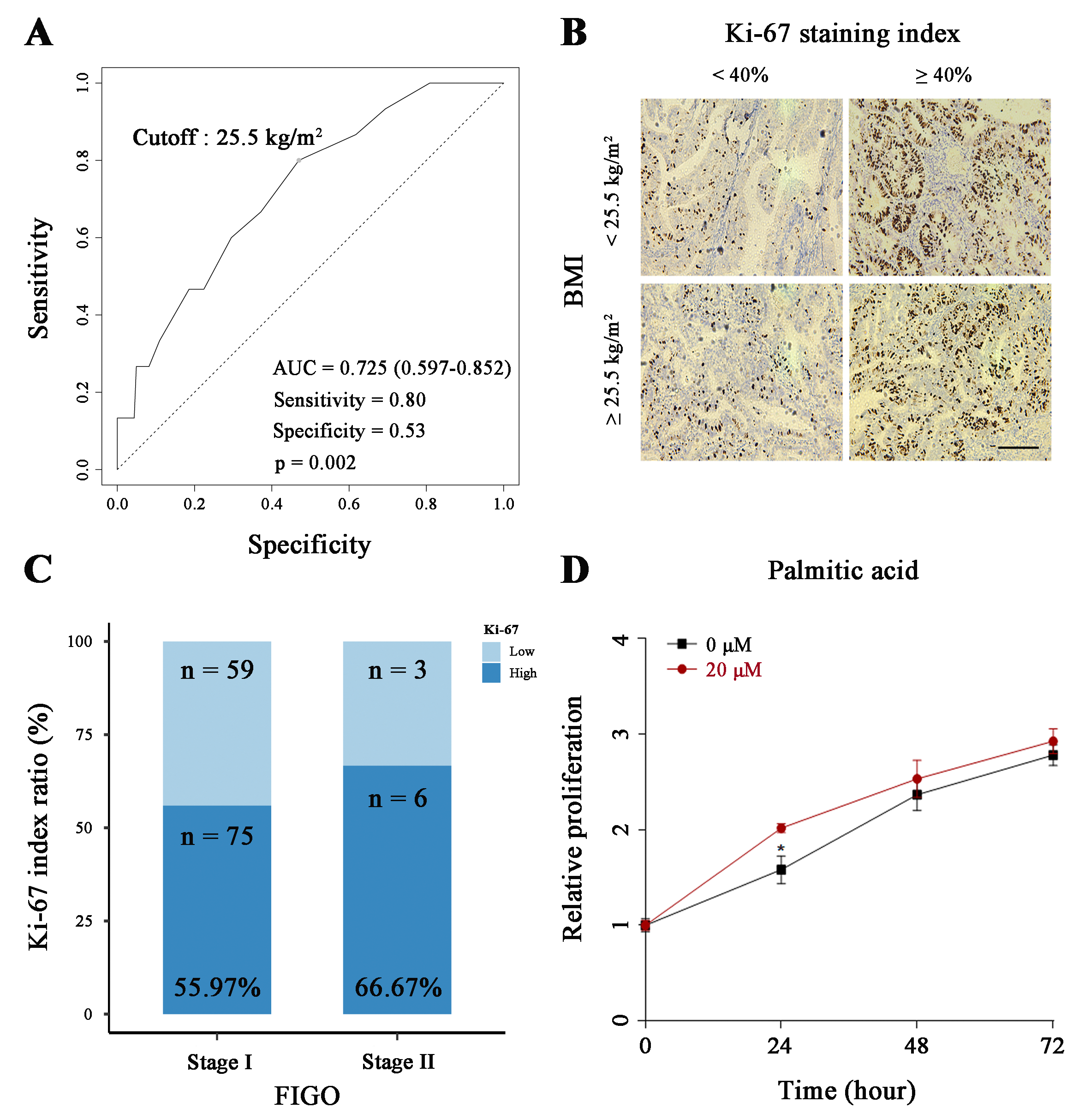

Supplement: Supplementary Figure S3 [file 26884844251374981_supplementary_figure_s3.tif]

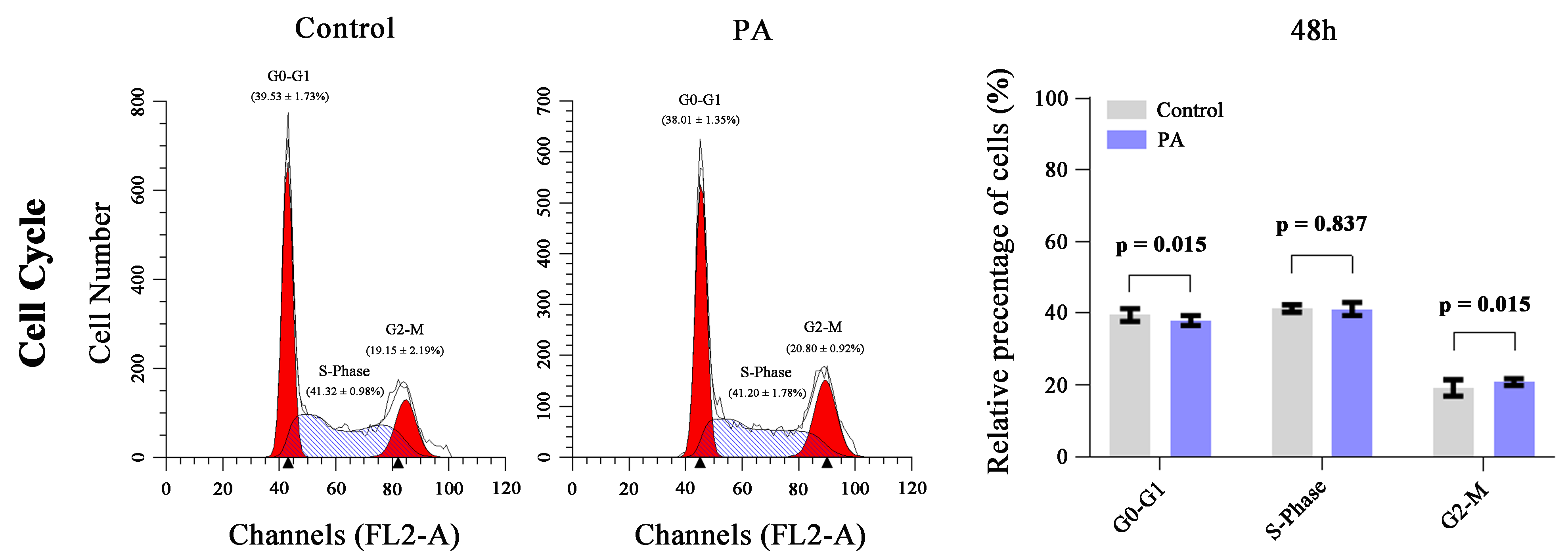

Supplement: Supplementary Figure S4 [file 26884844251374981_supplementary_figure_s4.tif]
