# Supplementary material for: Exploring the Impact of Obesity on Progression and Prognosis in Early-Stage Endometrioid Endometrial Carcinoma
Source: Womens Health Rep (New Rochelle). 2025 Sep 5;6(1):803–15. doi: 10.1177/26884844251374981 (PMC12528850; doi:10.1177/26884844251374981)
Supplement: Supplementary Table S1 [file 26884844251374981_supplementary_table_s1.docx]

**Supplemental Table 1.** **Mendelian randomization analysis of EEC and obesity.**

| **Method** | **No. of SNP** | **OR** | **95% CI** | **P - value** |
| --- | --- | --- | --- | --- |
| **Inverse variance weighted** | 2 | 3.540 x 10^13^ | 7.990 x 10^6^ - 1.568 x 10^20^ | **6.455 × 10^-5^** |
| **MR Egger** | - | - | - | - |
| **Weighted median** | - | - | - | - |
| **Simple mode** | - | - | - | - |
| **Weighted mode** | - | - | - | - |

***EEC****: Endometrioid endometrial carcinoma;* ***SNP****:* *Single nucleotide polymorphism;* ***OR****: Odds ratio;* ***CI****:* *Confidence interval*
