# Supplementary material for: Exploring the Impact of Obesity on Progression and Prognosis in Early-Stage Endometrioid Endometrial Carcinoma
Source: Womens Health Rep (New Rochelle). 2025 Sep 5;6(1):803–15. doi: 10.1177/26884844251374981 (PMC12528850; doi:10.1177/26884844251374981)
Supplement: Supplementary Table S2 [file 26884844251374981_supplementary_table_s2.docx]

**Supplemental Table 2. Mendelian randomization analysis of BMI and main components of adipocytes.**

| **Exposure variables** | **Method** | **No. of SNP** | **OR** | **95% CI** | **P - value** |
| --- | --- | --- | --- | --- | --- |
| **Monounsaturated fatty acids** | **Inverse variance weighted** | 51 | 1.110 | 0.905 - 1.361 | 0.316 |
|  | **MR Egger** | 51 | 1.173 | 0.835 - 1.649 | 0.363 |
|  | **Weighted median** | 51 | 1.033 | 0.746 - 1.431 | 0.845 |
|  | **Simple mode** | 51 | 0.806 | 0.428 - 1.518 | 0.508 |
|  | **Weighted mode** | 51 | 1.117 | 0.803 - 1.554 | 0.514 |
| **Polyunsaturated fatty acids** | **Inverse variance weighted** | 45 | 0.892 | 0.709 - 1.124 | 0.333 |
|  | **MR Egger** | 45 | 0.863 | 0.590 - 1.263 | 0.453 |
|  | **Weighted median** | 45 | 0.901 | 0.664 - 1.221 | 0.500 |
|  | **Simple mode** | 45 | 1.271 | 0.746 - 2.164 | 0.383 |
|  | **Weighted mode** | 45 | 0.933 | 0.686 - 1.268 | 0.658 |
| **Total phospholipid**  **level** | **Inverse variance weighted** | 55 | 1.030 | 0.838 - 1.266 | 0.777 |
|  | **MR Egger** | 55 | 0.836 | 0.568 - 1.232 | 0.370 |
|  | **Weighted median** | 55 | 0.991 | 0.731 - 1.343 | 0.954 |
|  | **Simple mode** | 55 | 0.936 | 0.559 - 1.567 | 0.801 |
|  | **Weighted mode** | 55 | 0.980 | 0.695 - 1.385 | 0.913 |
| **Total cholesterol**  **level** | **Inverse variance weighted** | 173 | 1.157 | 0.960 - 1.393 | 0.126 |
|  | **MR Egger** | 173 | 1.058 | 0.805 - 1.390 | 0.685 |
|  | **Weighted median** | 173 | 1.003 | 0.745 - 1.350 | 0.986 |
|  | **Simple mode** | 173 | 0.834 | 0.477 - 1.460 | 0.527 |
|  | **Weighted mode** | 173 | 1.051 | 0.808 - 1.367 | 0.711 |
| **LDL cholesterol**  **level** | **Inverse variance weighted** | 149 | 1.102 | 0.930 - 1.305 | 0.262 |
|  | **MR Egger** | 149 | 1.103 | 0.883 - 1.378 | 0.390 |
|  | **Weighted median** | 149 | 0.991 | 0.761 - 1.290 | 0.944 |
|  | **Simple mode** | 149 | 0.729 | 0.380 - 1.398 | 0.343 |
|  | **Weighted mode** | 149 | 1.026 | 0.817 - 1.287 | 0.827 |
| **HDL cholesterol**  **level** | **Inverse variance weighted** | 249 | 0.979 | 0.842 - 1.138 | 0.781 |
|  | **MR Egger** | 249 | 1.007 | 0.811 - 1.251 | 0.948 |
|  | **Weighted median** | 249 | 0.967 | 0.747 - 1.254 | 0.803 |
|  | **Simple mode** | 249 | 0.753 | 0.428 - 1.326 | 0.327 |
|  | **Weighted mode** | 249 | 0.967 | 0.786 - 1.189 | 0.747 |

***BMI****: Body mass index;* ***SNP****:Single nucleotide polymorphism;* ***OR****:Odds ratio;* ***CI****:* *Confidence interval;* ***LDL***: *Low density lipoprotein；****HDL****: High density lipoprotein*

***:***
