# Supplementary material for: Exploring the Impact of Obesity on Progression and Prognosis in Early-Stage Endometrioid Endometrial Carcinoma
Source: Womens Health Rep (New Rochelle). 2025 Sep 5;6(1):803–15. doi: 10.1177/26884844251374981 (PMC12528850; doi:10.1177/26884844251374981)
Supplement: Supplementary Table S3 [file 26884844251374981_supplementary_table_s3.docx]

**Supplemental Table 3.** **Clinical features of EEC patients from Clinical data.**

| **ID** | **Age** | **BMI（kg/m2）** | **CA125 (U/ml)** | **FPG**  **(mmol/L)** | **FIGO**  **stage** | **Grade** | **Lesion size (cm)** | **LVSI** | **P53 mutant** | **ER** | **PR** | **Ki67** | |
| --- | --- | --- | --- | --- | --- | --- | --- | --- | --- | --- | --- | --- | --- |
| BX001 | 82 | 16 | 14.53 | 4.02 | IA | unknown | 0.7 | - | unknown | unknown | unknown | unknown | |
| BX002 | 64 | 18 | 5.87 | 4.68 | IA | 3 | 4.7 | + | + | medium-strong (40%) | medium-strong (10%) | 60% | |
| BX003 | 64 | 18 | 16.75 | 4.68 | IA | unknown | 4.7 | unknown | + | medium-strong (40%) | medium-strong (10％) | 60% | |
| BX004 | 49 | 18 | 10.42 | 5.33 | IA | unknown | 4.5 | - | - | medium-strong (60%) | medium-strong (70%) | unknown | |
| BX005 | 57 | 18 | 45.63 | 5.62 | IB | unknown | 11 | + | + | + | unknown | unknown | |
| BX006 | 44 | 19 | 44.1 | 5.07 | unknown | unknown | unknown | - | - | medium-strong (75%) | strong (90%) | 15% | |
| BX007 | 53 | 20 | 24.47 | 5.2 | IB | unknown | 6 | + | - | strong (60%) | strong (30%) | 40% | |
| BX008 | 56 | 20 | 18.11 | 4.79 | IA | unknown | 4.5 | - | - | medium-strong (40%) | medium-strong (70%) | 50% | |
| BX009 | 57 | 20 | 12.17 | 5.24 | IA | unknown | 2 | - | unknown | strong (75%) | strong (75%) | 60% | |
| BX010 | 70 | 20 | 50.95 | 5.07 | IB | unknown | 7 | - | + | medium-strong (90%) | medium-strong (20%) | 60% | |
| BX011 | 57 | 20 | 169.8 | 4.77 | ⅢA | unknown | 9.5 | - | + | - | - | 70% | |
| BX012 | 50 | 20 | 17 | 4.92 | IA | unknown | unknown | - | unknown | unknown | unknown | unknown | |
| BX013 | 68 | 21 | 14.88 | 10.24 | IA | 1 | 1.2 | - | - | medium-strong (15%) | medium-strong (15%) | 12% | |
| BX014 | 82 | 21 | 13.38 | 6.28 | IA | unknown | 3 | unknown | unknown | medium-strong (70%) | medium-strong (80%) | 30% | |
| BX015 | 55 | 21 | 15.56 | 4.88 | IA | unknown | 6.5 | - | + | medium-strong (60%) | medium-strong (60%) | 30% | |
| BX016 | 50 | 21 | 34.93 | 4.45 | IA | unknown | unknown | - | - | medium-strong (90%) | medium-strong (90%) | 30% | |
| BX017 | 61 | 21 | unknown | 5.96 | IB | unknown | unknown | - | unknown | medium-strong (50%) | strong (50%) | 30% | |
| BX018 | 59 | 21 | unknown | 5.04 | IA | 0 | 2.5 | - | - | medium-strong (80%) | medium-strong (60%) | 40% | |
| BX019 | 61 | 21 | 7.63 | 6.08 | IA | unknown | 4 | unknown | + | - | - | 40% | |
| BX020 | 57 | 21 | 22.09 | 4.81 | IA | unknown | 3.5 | - | unknown | medium-strong (60%) | medium-strong (50%) | 50% | |
| BX021 | 46 | 21 | 23.3 | 4.68 | IA | unknown | 3 | + | - | medium (15%) | strong (40%) | 35% | |
| BX022 | 49 | 21 | 24.85 | 4.56 | IA | unknown | unknown | - | unknown | weak-medium (40%) | weak-medium (40%) | 60% | |
| BX023 | 54 | 21 | 158.2 | 4.94 | ⅢC | 1 | 9 | + | - | medium-strong (80%) | unknown | 70% | |
| BX024 | 62 | 21 | 10.08 | 5.14 | IB | unknown | 1.7 | + | unknown | medium-strong (80%) | - | 30% | |
| BX025 | 47 | 21 | 25.5 | 4.91 | IA | unknown | 2.5 | - | unknown | unknown | unknown | unknown | |
| BX026 | 51 | 21 | 19.41 | 5.24 | IA | unknown | 3 | unknown | + | medium-strong (90%) | medium (90%) | unknown | |
| BX027 | 62 | 21 | 19.03 | 5.85 | IIIC | 2 | 3.5 | + | - | - | - | unknown | |
| BX028 | 58 | 22 | 275 | 5.25 | IA | unknown | 4.5 | - | - | medium-strong (70%) | medium-strong (70%) | 25% | |
| BX029 | 58 | 22 | 14.62 | 6.42 | IA | 0 | 0.8 | - | - | medium-strong (80%) | strong (60%) | 30% | |
| BX030 | 49 | 22 | 13.16 | 5.16 | IA | unknown | 2.8 | - | unknown | medium-strong (70%) | medium-strong (80%) | 35% | |
| BX031 | 49 | 22 | 13.87 | 5.16 | IA | 1 | 2.8 | - | unknown | medium-strong (70%) | medium-strong (80%) | 35% | |
| BX032 | 51 | 22 | 78.68 | 6.18 | IA | unknown | 5 | unknown | unknown | strong (90%) | strong (90%) | 40% | |
| BX033 | 57 | 22 | 14.28 | 5.84 | IA | 2 | 2.5 | - | - | medium (20%) | weak-medium (2%) | 25% | |
| BX034 | 57 | 22 | 30.28 | 5.84 | IA | unknown | 2.5 | - | - | medium (20%) | weak-medium (2%) | 40% | |
| BX035 | 61 | 22 | 21.22 | 4.66 | IA | unknown | 3 | - | - | strong (20%) | strong (30%) | 20% | |
| BX036 | 62 | 22 | unknown | unknown | IA | unknown | 4.7 | + | - | - | - | 50% | |
| BX037 | 59 | 22 | 8.69 | 4.78 | IA | unknown | 3 | - | unknown | strong (70%) | strong (40%) | 30% | |
| BX038 | 58 | 22 | 20.94 | 5.3 | IA | unknown | 2.5 | - | unknown | unknown | unknown | unknown | |
| BX039 | 63 | 22 | 23.2 | 7.65 | IA | 2 | 4.5 | + | - | medium-strong (95%) | strong (95%) | unknown | |
| BX040 | 63 | 22 | 220.8 | 5.82 | IV | unknown | unknown | + | unknown | unknown | unknown | unknown | |
| BX041 | 56 | 22 | 113.1 | 15.51 | unknown | unknown | unknown | unknown | unknown | unknown | unknown | unknown | |
| BX042 | 65 | 23 | 33.07 | 4.48 | IA | unknown | 6 | - | unknown | medium-strong (55%) | medium-strong (55%) | 8% | |
| BX043 | 41 | 23 | 23.1 | 7.29 | IA | unknown | 4 | - | - | + | + | 10% | |
| BX044 | 59 | 23 | 42.86 | 6.48 | IB | unknown | unknown | + | - | medium-strong (80%) | strong (80%) | 20% | |
| BX045 | 65 | 23 | 13.96 | 4.66 | IA | unknown | 2.2 | - | unknown | medium-strong (90%) | medium-strong (20%) | 30% | |
| BX046 | 65 | 23 | 11.55 | 5.68 | IA | 1 | 2 | + | - | medium-strong (80%) | medium-strong (80%) | 30% | |
| BX047 | 31 | 23 | 34.38 | 4.6 | IIIA | unknown | 4 | - | - | medium-strong (70%) | medium-strong (70%) | 30% | |
| BX048 | 52 | 23 | 11.73 | unknown | IIIC | 3 | 4.5 | + | - | - | - | 30% | |
| BX049 | 57 | 23 | 8.38 | 5.51 | IA | 1 | 3.2 | - | unknown | medium-strong (60%) | medium-strong (80%) | 35% | |
| BX050 | 54 | 23 | 112.9 | 4.73 | IA | 0 | 1.5 | - | unknown | medium-strong (70%) | medium-strong (60%) | 35% | |
| BX051 | 57 | 23 | 11.82 | 5.51 | unknown | unknown | 3.2 | unknown | unknown | medium-strong (60%) | medium-strong (80%) | 35% | |
| BX052 | 55 | 23 | 12.23 | 4.87 | IA | unknown | 3 | - | + | medium-strong (80%) | medium-strong (70%) | 25% | |
| BX053 | 51 | 23 | 16.46 | 4.72 | IA | unknown | 2 | - | - | medium-strong (90%) | medium-strong (80%) | 40% | |
| BX054 | 45 | 23 | 4.51 | unknown | IA | 2 | 4.5 | - | unknown | weak-medium (70%) | medium-strong (90%) | 40% | |
| BX055 | 52 | 23 | 20.1 | 5.34 | IA | 0 | 3.5 | - | - | weak-medium (15%) | medium-strong (90%) | 30% | |
| BX056 | 55 | 23 | 25.66 | 4.86 | IA | unknown | 2 | - | - | medium-strong (70%) | medium-strong (60%) | 50% | |
| BX057 | 57 | 23 | unknown | 8.11 | IB | unknown | 1.5 | - | - | medium (10%) | strong (40%) | 20% | |
| BX058 | 49 | 23 | 17.34 | 4.84 | IA | unknown | 2 | - | + | weak-medium (30％) | + | 60% | |
| BX059 | 53 | 23 | 25.7 | 10.84 | IA | 0 | 5.5 | + | - | strong (80%) | strong (40%) | 70% | |
| BX060 | 55 | 23 | 4.9 | 4.58 | IB | unknown | 0.5 | unknown | unknown | medium-strong (50%) | medium-strong (40%) | 45% | |
| BX061 | 63 | 23 | 8.08 | 5.49 | IA | 1 | 2.2 | - | unknown | medium-strong (80%) | medium-strong (80%) | unknown | |
| BX062 | 52 | 23 | 51.8 | 4.78 | IA | 0 | 1.4 | - | unknown | unknown | unknown | unknown | |
| BX063 | 57 | 23 | 13.44 | 4.96 | IA | unknown | 0.7 | unknown | unknown | unknown | unknown | unknown | |
| BX064 | 49 | 23 | 18.53 | 4.68 | IA | unknown | 2.3 | unknown | unknown | unknown | unknown | unknown | |
| BX065 | 46 | 23 | 29 | 5.42 | IA | unknown | unknown | unknown | unknown | unknown | unknown | unknown | |
| BX066 | 38 | 23 | 7.58 | 5.13 | II | unknown | unknown | - | unknown | unknown | unknown | unknown | |
| BX067 | 45 | 23 | 5.9 | 4.49 | unknown | unknown | 1.1 | unknown | unknown | unknown | unknown | unknown | |
| BX068 | 69 | 23 | 10.59 | 4.82 | unknown | unknown | unknown | unknown | unknown | unknown | unknown | unknown | |
| BX069 | 55 | 24 | 10.65 | 5.77 | IA | unknown | 6.5 | - | - | medium-strong (90%) | medium (85%) | 20% | |
| BX070 | 53 | 24 | 17.72 | 5.16 | IA | unknown | 2.5 | - | - | medium-strong (40%) | medium-strong (50%) | 20% | |
| BX071 | 50 | 24 | 13.3 | 4.95 | unknown | unknown | unknown | - | + | medium-strong (90%) | medium-strong (90%) | 20% | |
| BX072 | 68 | 24 | 32.3 | 6.3 | IA | 2 | 5.9 | unknown | - | medium-strong (80%) | strong (80%) | 30% | |
| BX073 | 44 | 24 | 39.61 | 4.66 | unknown | unknown | 2.8 | - | + | medium-strong (50%) | medium-strong (50%) | 32% | |
| BX074 | 64 | 24 | 37.81 | 5.53 | IA | unknown | 3 | unknown | unknown | medium-strong (30%) | medium-strong (40%) | 35% | |
| BX075 | 58 | 24 | 12.19 | 5.34 | IA | unknown | 3.6 | + | - | medium-strong (90%) | medium-strong (90%) | 40% | |
| BX076 | 56 | 24 | 15.27 | 7.23 | IA | 1 | 1.1 | - | - | medium-strong (60%) | medium (40%) | 40% | |
| BX077 | 55 | 24 | unknown | 5.09 | IA | 2 | 3.1 | - | unknown | medium-strong (40%) | strong (70%) | 40% | |
| BX078 | 69 | 24 | 9.8 | 5.48 | IA | unknown | 2.4 | - | - | medium-strong (70%) | medium-strong (80%) | 60% | |
| BX079 | 65 | 24 | 16.54 | 7.57 | IA | 2 | 9.5 | + | + | strong (90%) | strong (45%) | 65% | |
| BX080 | 42 | 24 | 27.3 | 5.97 | ⅡC | unknown | 5 | + | - | - | - | 80% | |
| BX081 | 57 | 24 | 66.66 | 5.66 | IA | unknown | 1.5 | + | unknown | unknown | unknown | unknown | |
| BX082 | 54 | 24 | 33.3 | 7.2 | IA | 1 | 1.5 | - | unknown | unknown | unknown | unknown | |
| BX083 | 65 | 24 | 10.9 | 5.48 | IA | 0 | 9.5 | + | - | strong (90%) | strong (45%) | unknown | |
| BX084 | 65 | 24 | 17.67 | 5.59 | IA | 0 | 1 | - | unknown | unknown | unknown | unknown | |
| BX085 | 61 | 24 | 12.71 | 6.85 | IA | unknown | 3.5 | + | unknown | unknown | unknown | unknown | |
| BX086 | 54 | 24 | 103.8 | 6.72 | IIIC | 1 | 3.5 | + | unknown | unknown | unknown | unknown | |
| BX087 | 47 | 25 | 11.61 | 4.62 | IIA | unknown | 9 | + | - | medium-strong (70%) | strong (90%) | 15% | |
| BX088 | 46 | 25 | 9.24 | 6.19 | IA | 2 | 7 | - | unknown | medium (60%) | weak (70%) | 20% | |
| BX089 | 80 | 25 | 24.82 | 5.54 | IA | unknown | 2.2 | - | unknown | strong (60%) | strong (25%) | 30% | |
| BX090 | 51 | 25 | 5 | 13.12 | IA | 2 | 7 | - | unknown | medium-strong (80%) | medium-strong (80%) | 30% | |
| BX091 | 70 | 25 | 14.03 | 6.04 | IA | unknown | 1 | - | - | medium-strong (30%) | medium-strong (40%) | 30% | |
| BX092 | 76 | 25 | 29.96 | 7.17 | IB | unknown | 2.5 | - | - | strong (70%) | strong (70%) | 30% | |
| BX093 | 62 | 25 | 14.5 | unknown | IB | 1 | 3.3 | - | unknown | strong (90%) | strong (90%) | 30% | |
| BX094 | 58 | 25 | 13.77 | 5.98 | IB | unknown | 3.5 | - | unknown | medium-strong (50%) | medium-strong (50%) | 35% | |
| BX095 | 45 | 25 | 14.58 | 5.23 | IA | unknown | 4.5 | - | unknown | weak-medium (70%) | medium-strong (90%) | 40% | |
| BX096 | 59 | 25 | 57.2 | 6.77 | IA | 0 | 6.5 | - | - | medium-strong (90%) | medium-strong (90%) | 40% | |
| BX097 | 55 | 25 | 5.2 | 5.43 | IA | unknown | 1.5 | unknown | unknown | medium-strong (40%) | strong (30%) | 40% | |
| BX098 | 38 | 25 | 14.09 | 5.4 | IA | unknown | 1.8 | - | - | medium-strong (90%) | medium-strong (90%) | 40% | |
| BX099 | 57 | 25 | 22.97 | 4.31 | IA | unknown | 3.2 | - | unknown | medium-strong (20%) | medium-strong (40%) | 50% | |
| BX100 | 57 | 25 | 11.14 | unknown | IA | 3 | 3.2 | - | unknown | medium-strong (20%) | medium-strong (50%) | 50% | |
| BX101 | 46 | 25 | 6.13 | unknown | IB | 3 | 7 | + | + | weak-medium (20%) | medium-strong (50%) | 50% | |
| BX102 | 55 | 25 | 26.15 | 5.32 | IA | unknown | 2.8 | - | - | strong (90%) | strong (90%) | 55% | |
| BX103 | 62 | 25 | 29.04 | 5.35 | IA | unknown | 1 | - | unknown | medium (30%) | weak-medium (35%) | 55% | |
| BX104 | 62 | 25 | 141.2 | 5.94 | IB | unknown | 3.5 | - | - | medium-strong (90%) | medium-strong (70%) | 60% | |
| BX105 | 64 | 25 | 101.5 | 7.57 | ⅢA | unknown | 7 | + | - | medium-strong (70%) | medium-strong (80%) | 70% | |
| BX106 | 61 | 25 | 38.24 | 8.25 | IA | unknown | 2.5 | - | + | strong (40%) | strong (5%) | 70% | |
| BX107 | 64 | 25 | 445.20 | 5.16 | IV | unknown | unknown | + | + | + | - | 80% | |
| BX108 | 67 | 25 | 25.6 | 6.09 | IA | 0 | 4.5 | + | unknown | unknown | unknown | unknown | |
| BX109 | 57 | 25 | 14.09 | 7.24 | IA | unknown | 3.5 | - | unknown | unknown | unknown | unknown | |
| BX110 | 46 | 25 | 19.53 | 6.19 | IA | unknown | 7 | - | unknown | unknown | unknown | unknown | |
| BX111 | 61 | 25 | 18.1 | 5.82 | IA | 1 | unknown | unknown | unknown | unknown | unknown | unknown | |
| BX112 | 51 | 25 | 22.9 | 5.95 | IA | unknown | 7 | - | unknown | unknown | unknown | unknown | |
| BX113 | 50 | 25 | 37.93 | 5.03 | IA | unknown | 2.2 | - | unknown | unknown | unknown | unknown | |
| BX114 | 54 | 25 | 18.26 | 5.21 | IA | unknown | unknown | - | unknown | unknown | unknown | unknown | |
| BX115 | 62 | 25 | 7.43 | 7.12 | IB | unknown | 3.3 | - | unknown | unknown | unknown | unknown | |
| BX116 | 56 | 25 | 4.57 | 5.6 | IB | unknown | 3.5 | - | unknown | unknown | unknown | unknown | |
| BX117 | 56 | 26 | 6.94 | 5.44 | IA | 1 | 4.5 | - | - | medium (10%) | medium-strong (40%) | 25% | |
| BX118 | 56 | 26 | 8.2 | 5.67 | IA | 0 | 2.5 | - | + | medium-strong (80%) | medium-strong (80%) | 35% | |
| BX119 | 66 | 26 | 5.83 | 5.59 | IA | unknown | 3 | - | - | medium-strong (80%) | strong (80%) | 40% | |
| BX120 | 55 | 26 | 14.77 | 5.71 | IC | unknown | 2.5 | - | + | strong(65%) | strong (60%) | 40% | |
| BX121 | 35 | 26 | 14.34 | 4.66 | IA | unknown | 3 | - | - | medium-strong (70%) | strong (70%) | 45% | |
| BX122 | 63 | 26 | 13.06 | unknown | IB | 2 | 3.5 | + | - | strong (60%) | strong (70%) | 45% | |
| BX123 | 84 | 26 | unknown | 7.35 | IA | 2 | 5.2 | - | - | medium-strong (80%) | medium-strong (70%) | 50% | |
| BX124 | 46 | 26 | 6.13 | 6.06 | IB | unknown | 7 | unknown | + | medium (20%) | medium-strong (50%) | 50% | |
| BX125 | 55 | 26 | 4.38 | 5.23 | IB | 2 | 3.2 | - | unknown | medium-strong (70%) | strong (70%) | 50% | |
| BX126 | 55 | 26 | 95.84 | 5.23 | IB | unknown | 3.2 | - | unknown | medium-strong (70%) | strong (70%) | 50% | |
| BX127 | 50 | 26 | 4 | 5.69 | II | unknown | 5 | + | - | + | unknown | 50% | |
| BX128 | 57 | 26 | 38.69 | 6.9 | IIIC | unknown | unknown | + | - | strong (90%) | medium (90%) | 50% | |
| BX129 | 84 | 26 | 20.45 | 7.35 | unknown | unknown | 5.2 | - | - | medium-strong (80%) | medium-strong (70%) | 50% | |
| BX130 | 57 | 26 | 20.65 | 14.36 | IA | unknown | 3 | - | + | weak-medium (30%) | medium-strong (60%) | 55% | |
| BX131 | 58 | 26 | unknown | unknown | IA | 2 | 4.5 | - | + | + | + | 70% | |
| BX132 | 49 | 26 | 16.5 | 4.41 | ⅡA | unknown | 3.5 | - | unknown | unknown | unknown | unknown | |
| BX133 | 57 | 26 | 4.57 |  | IA | unknown | 1.6 | - | unknown | unknown | unknown | unknown | |
| BX134 | 62 | 26 | 46.18 | 5.45 | IA | unknown | 5 | - | unknown | medium-strong (60%) | medium-strong (40%) | unknown | |
| BX135 | 68 | 26 | 29.46 | 6.62 | IA | unknown | 1.3 | unknown | unknown | unknown | unknown | unknown | |
| BX136 | 46 | 26 | 9.83 | 4.89 | IA | unknown | unknown | unknown | unknown | unknown | unknown | unknown | |
| BX137 | 72 | 26 | 9.12 | 5.84 | IB | unknown | 4.2 | - | - | medium-strong (90%) | medium-strong (70%) | unknown | |
| BX138 | 63 | 26 | 16.6 | 6.18 | IB | unknown | 3.5 | + | unknown | unknown | unknown | unknown | |
| BX139 | 61 | 26 | 14.7 | 6.05 | IIIB | unknown | unknown | + | unknown | unknown | unknown | unknown | |
| BX140 | 66 | 27 | unknown | 3.96 | IA | unknown | 2 | unknown | - | medium (10%) | strong (90%) | 20% | |
| BX141 | 46 | 27 | 18.5 | 8.67 | unknown | unknown | 1.5 | - | - | medium-strong (90%) | medium-strong (90%) | 20% | |
| BX142 | 61 | 27 | 14.03 | 6.49 | IB | unknown | 3 | - | unknown | medium-strong (80%) | medium-strong (90%) | 30% | |
| BX143 | 69 | 27 | 9.53 | 5.25 | IA | unknown | 2.5 | + | - | medium-strong (90%) | medium-strong (30%) | 35% | |
| BX144 | 59 | 27 | 12.33 | 5.07 | IA | unknown | 1.4 | - | - | strong (90%) | strong (90%) | 35% | |
| BX145 | 61 | 27 | 26.12 | 6.93 | IA | unknown | 2 | - | unknown | medium-strong (70%) | medium-strong (85%) | 37% | |
| BX146 | 62 | 27 | 23.59 | 5.22 | IA | unknown | 3.5 | + | - | medium-strong (80%) | medium-strong (60%) | 40% | |
| BX147 | 58 | 27 | unknown | 6.15 | IA | 1 | 1.8 | - | unknown | strong (80%) | strong (70%) | 40% | |
| BX148 | 59 | 27 | 9.42 | 5.41 | IA | unknown | unknown | - | - | medium-strong (90%) | medium-strong (90%) | 55% | |
| BX149 | 65 | 27 | 16.26 | 7.3 | IA | unknown | 3 | - | - | strong (80%) | strong (70%) | 60% | |
| BX150 | 55 | 27 | 74.85 | 5.17 | IA | unknown | 3.2 | - | unknown | strong (90%) | strong (80%) | 65% | |
| BX151 | 56 | 27 | 32.76 | 5.08 | IIIC | 1 | 7 | + | - | weak-medium (5%) | weak-medium (3%) | 75% | |
| BX152 | 54 | 27 | 15.5 | 5.54 | Ⅱ | unknown | 4.5 | + | + | strong (80%) | strong (90%) | 80% | |
| BX153 | 62 | 27 | 7.3 | 5.25 | IA | 2 | 2.5 | - | unknown | unknown | unknown | unknown | |
| BX154 | 53 | 27 | unknown | unknown | IA | unknown | 3 | + | unknown | unknown | unknown | unknown | |
| BX155 | 58 | 27 | 42.2 | 6.44 | IA | 0 | 4.5 | - | unknown | unknown | unknown | unknown | |
| BX156 | 51 | 27 | 10.28 | 5.04 | IA | unknown | unknown | - | unknown | unknown | unknown | unknown | |
| BX157 | 39 | 27 | unknown | unknown | unknown | unknown | unknown | - | - | medium-strong (80%) | medium-strong (70%) | unknown | |
| BX158 | 66 | 28 | unknown | 5.95 | IA | unknown | unknown | - | - | medium-strong (75%) | medium-strong (90%) | 10% | |
| BX159 | 54 | 28 | 15.08 | 6.7 | IA | unknown | 2 | - | - | medium (80%) | medium-strong (80%) | 15% | |
| BX160 | 70 | 28 | unknown | 5.1 | IB | 2 | 2 | - | unknown | strong (90%) | strong (90%) | 20% | |
| BX161 | 90 | 28 | 18.25 | 5.1 | IB | unknown | 2 | - | unknown | strong (90%) | strong (90%) | 20% | |
| BX162 | 66 | 28 | 25.65 | 4.95 | IIIB | unknown | 4.5 | + | - | + | unknown | 20% | |
| BX163 | 62 | 28 | 18.83 | 5.46 | IA | unknown | 2 | unknown | unknown | medium-strong (60%) | medium-strong (90%) | 30% | |
| BX164 | 61 | 28 | 14.84 | 6.36 | IA | unknown | 4 | + | - | medium-strong (70%) | strong (80%) | 30% | |
| BX165 | 61 | 28 | 6.48 | 6.36 | IA | 2 | 2 | - | - | strong (70%) | strong (80%) | 30% | |
| BX166 | 65 | 28 | 9.76 | 5.79 | IA | unknown | 2.3 | + | + | medium-strong (90%) | medium-strong (80%) | 40% | |
| BX167 | 58 | 28 | 26.2 | 5.47 | IA | 0 | 1.7 | - | - | weak-medium (20%) | medium-strong (40%) | 50% | |
| BX168 | 56 | 28 | 62.08 | 5.17 | IB | unknown | 3.4 | - | unknown | strong (80%) | strong (70%) | 55% | |
| BX169 | 53 | 28 | 12.84 | 6.41 | II | unknown | 4.8 | - | unknown | weak-medium (40%) | medium-strong (80%) | 55% | |
| BX170 | 58 | 28 | 44.5 | 14.4 | IA | 2 | 4 | + | unknown | medium-strong (90%) | medium-strong (90%) | 90% | |
| BX171 | 60 | 28 | 206.6 | 6.61 | IA | 2 | 10.3 | + | unknown | unknown | unknown | unknown | |
| BX172 | 51 | 28 | 97.49 | 6.92 | IA | unknown | 2 | - | unknown | unknown | unknown | unknown |  |
| BX173 | 53 | 28 | 29.47 | 6.57 | II | unknown | 2.5 | - | unknown | unknown | unknown | unknown |  |
| BX174 | 54 | 28 | 40.64 | 8.92 | unknown | unknown | unknown | unknown | unknown | unknown | unknown | unknown |  |
| BX175 | 85 | 28 | 16.49 | 5.76 | unknown | unknown | unknown | unknown | unknown | unknown | unknown | unknown |  |
| BX176 | 67 | 29 | 22.75 | 5.85 | unknown | unknown | unknown | unknown | unknown | + | + | 15% |  |
| BX177 | 65 | 29 | 14.44 | 12.32 | IA | unknown | 0.4 | - | unknown | medium-strong (70%) | medium-strong (90%) | 20% |  |
| BX178 | 49 | 29 | unknown | 5.56 | IA | 0 | 2 | - | - | medium-strong (60%) | medium-strong (60%) | 35% |  |
| BX179 | 64 | 29 | 12.08 | 7.29 | IA | unknown | 1 | - | - | medium-strong (80%) | - | 40% |  |
| BX180 | 73 | 29 | unknown | 5.51 | IA | unknown | 3.5 | - | unknown | strong (60%) | strong (60%) | 45% |  |
| BX181 | 41 | 29 | 40.19 | 5.85 | IA | unknown | 3.5 | - | - | medium (80%) | medium-strong (80%) | 55% |  |
| BX182 | 53 | 29 | 9.1 | 5.57 | IA | unknown | 1.5 | - | + | strong (90%) | strong (90%) | 60% |  |
| BX183 | 68 | 29 | 43.51 | 5.86 | IB | unknown | 3.5 | - | unknown | unknown | unknown | unknown |  |
| BX184 | 39 | 30 | 35.68 | 5.53 | IA | unknown | unknown | - | unknown | medium-strong (60%) | medium-strong (80%) | 15% |  |
| BX185 | 57 | 30 | 34.1 | 5.45 | IA | 0 | 0.9 | + | - | medium-strong (60%) | medium-strong (60%) | 20% |  |
| BX186 | 65 | 30 | 39.87 | 11.83 | IA | unknown | 2.5 | - | - | strong (90%) | strong (90%) | 60% |  |
| BX187 | 64 | 30 | 32.2 | 7.05 | IB | 0 | 6 | + | + | medium-strong (65%) | strong (65%) | 70% |  |
| BX188 | 67 | 30 | 17.57 | 5.57 | IB | unknown | 3.3 | + | - | weak-medium (50%) | medium-strong (20%) | 45% |  |
| BX189 | 67 | 30 | 13.52 | 5.18 | IA | unknown | 2 | - | - | medium-strong (70%) | medium-strong (50%) | 40% |  |
| BX190 | 54 | 30 | 5.53 | 6.29 | II | 3 | 2.6 | + | - | strong (60%) | strong (60%) | 40% |  |
| BX191 | 54 | 30 | 7.73 | 6.29 | II | unknown | 2.6 | + | - | strong (60%) | strong (60%) | 40% |  |
| BX192 | 62 | 30 | 66.54 | 5.12 | IB | unknown | 5.5 | + | - | medium-strong (70%) | medium-strong (70%) | 50% |  |
| BX193 | 67 | 30 | 608.5 | 10.4 | IB | unknown | 4.1 | - | - | medium-strong (90%) | strong (75%) | 50% |  |
| BX194 | 53 | 30 | 23.8 | 5.69 | IA | 0 | 2 | - | - | medium-strong (80%) | strong (90%) | 70% |  |
| BX195 | 49 | 30 | 12.62 | 4.54 | I | unknown | 2.6 | - | - | medium-strong (80%) | medium-strong (80%) | unknown |  |
| BX196 | 66 | 30 | 20.4 | 5.9 | IA | 1 | 1.8 | - | unknown | unknown | unknown | unknown |  |
| BX197 | 53 | 30 | 14 | 5.61 | IA | 0 | 4 | - | unknown | unknown | unknown | unknown |  |
| BX198 | 72 | 30 | unknown | 7.5 | IA | 2 | 4 | - | unknown | unknown | unknown | unknown |  |
| BX199 | 64 | 30 | 14.1 | 7.13 | IA | 2 | 4.5 | unknown | unknown | unknown | unknown | unknown |  |
| BX200 | 63 | 31 | 26.8 | 6.49 | IA | unknown | 5 | - | - | strong (15%) | strong (15%) | 10% |  |
| BX201 | 54 | 31 | 44.7 | 8085 | IA | unknown | 1.5 | unknown | unknown | strong (90%) | strong (90%) | 20% |  |
| BX202 | 37 | 31 | 39.5 | 7.22 | IA | 0 | 3 | - | unknown | medium-strong (20%) | strong (30%) | 50% |  |
| BX203 | 61 | 31 | 53.7 | 6.2 | IIB | unknown | 4.5 | unknown | - | medium-strong (90%) | strong (95%) | 30% |  |
| BX204 | 59 | 31 | 496.3 | 5.62 | IIIC | 1 | 7.5 | + | + | - | - | 30% |  |
| BX205 | 46 | 31 | 19.6 | 4.5 | IA | unknown | 1.2 | - | - | medium-strong (50%) | medium-strong (50%) | 35% |  |
| BX206 | 65 | 31 | 117.7 | 7.86 | IA | unknown | 6 | - | - | weak-medium (20%) | medium-strong (20%) | 55% |  |
| BX207 | 40 | 31 | 22.45 | 5.30 | unknown | unknown | 1.2 | unknown | unknown | unknown | unknown | unknown |  |
| BX208 | 48 | 31 | unknown | 7.1 | unknown | unknown | unknown | unknown | unknown | unknown | unknown | unknown |  |
| BX209 | 30 | 31 | 128 | 5.11 | unknown | unknown | unknown | unknown | unknown | unknown | unknown | unknown |  |
| BX210 | 57 | 32 | 20.98 | 5.78 | IA | 1 | 2 | - | unknown | medium-strong (20%) | medium-strong (30%) | 20% |  |
| BX211 | 65 | 32 | 31.12 | 7.97 | IA | unknown | 3 | - | - | medium (70%) | strong (80%) | 55% |  |
| BX212 | 52 | 32 | 34.4 | 4.91 | IA | 0 | 5 | - | + | medium (80%) | medium-strong (80%) | 60% |  |
| BX213 | 62 | 32 | 3.59 | 4.99 | IB | unknown | 6 | + | - | medium-strong (80%) | medium-strong (70%) | 60% |  |
| BX214 | 61 | 32 | 20.69 | 6.57 | IA | unknown | 3 | - | unknown | unknown | unknown | unknown |  |
| BX215 | 51 | 32 | 15.23 | 4.86 | IA | unknown | unknown | - | unknown | unknown | unknown | unknown |  |
| BX216 | 52 | 33 | 36.41 | 7.53 | IA | 1 | 2 | - | unknown | medium (80%) | medium-strong (90%) | 40% |  |
| BX217 | 41 | 33 | 13.9 | 5.1 | Ⅱ | unknown | 2.3 | + | unknown | unknown | unknown | unknown |  |
| BX218 | 41 | 33 | 13.9 | 5.1 | Ⅱ | unknown | unknown | - | unknown | unknown | unknown | unknown |  |
| BX219 | 35 | 34 | 11.2 | 7.2 | IA | 0 | 8 | unknown | unknown | unknown | unknown | unknown |  |
| BX220 | 51 | 37 | 7.03 | 4.9 | IA | unknown | 2.6 | - | - | medium-strong (80%) | strong (95%) | 20% |  |
| BX221 | 49 | 37 | 11.51 | 5.32 | IA | 1 | 5.5 | - | unknown | medium-strong (70%) | medium-strong (80%) | 40% |  |
| BX222 | 49 | 37 | 18.16 | 5.32 | unknown | unknown | unknown | unknown | unknown | unknown | unknown | unknown |  |
| BX223 | 39 | 38 | 11.88 | 7.85 | IA | 2 | unknown | - | + | medium-strong (70%) | medium-strong (90%) | 20% |  |
| BX224 | 41 | 38 | 13.61 | 10.85 | IA | unknown | 7.2 | - | - | weak (40%) | weak-medium (80%) | unknown |  |
| BX225 | 56 | 39 | 42.29 | 9.77 | IA | 2 | 3.7 | - | + | medium-strong (50%) | medium-strong (80%) | 60% |  |
| BX226 | 56 | 39 | 63.76 | 9.77 | unknown | unknown | 3.7 | - | + | medium-strong (50%) | medium-strong (80%) | 30% |  |
| BX227 | 49 | 40 | 12.09 | 5.51 | IA | unknown | 6.5 | - | unknown | medium-strong (65%) | medium-strong (90%) | 45% |  |
| BX228 | 49 | 40 | 7.22 | 5.51 | IA | 1 | 6.5 | - | unknown | medium-strong (65%) | medium-strong (90%) | 40% |  |
| BX229 | 54 | 41 | 9.03 | 5.32 | II | unknown | 3.5 | - | unknown | medium-strong (95%) | medium-strong (60%) | 15% |  |
| BX230 | 57 | 42 | unknown | 4.92 | unknown | unknown | 2.5 | - | - | medium-strong (70%) | medium-strong (70%) | 20% |  |
| BX231 | 67 | 83 | 14.28 | 5.76 | II | unknown | unknown | + | - | - | - | unknown |  |

***EEC****: endometrioid endometrial carcinoma;* ***FPG****: Fasting plasma glucose;* ***LVSI****: Lymphovascular space invasion;* ***ER****: Estrogen receptor;* ***PR****: Progesterone receptor*
